# Evolution of the Quorum Sensing Regulon in Cooperating Populations of Pseudomonas aeruginosa

**DOI:** 10.1128/mbio.00161-22

**Published:** 2022-02-22

**Authors:** Nicole E. Smalley, Amy L. Schaefer, Kyle L. Asfahl, Crystal Perez, E. Peter Greenberg, Ajai A. Dandekar

**Affiliations:** a Department of Medicine, University of Washington, Seattle, Washington, USA; b Department of Microbiology, University of Washington, Seattle, Washington, USA; Georgia Institute of Technology School of Biological Sciences

**Keywords:** acyl-homoserine lactone, adaptive evolution, metatranscriptomics, quorum quenching

## Abstract

In the opportunistic pathogenic bacterium Pseudomonas aeruginosa acyl-homoserine lactone quorum sensing (QS) can activate expression of dozens to hundreds of genes depending on the strain under investigation. Many QS-activated genes code for extracellular products. P. aeruginosa has become a model for studies of cell-cell communication and coordination of cooperative activities, which result from production of extracellular products. We hypothesized that strain variation in the size of the QS regulon might reflect the environmental history of an isolate. We tested the hypothesis by performing long-term growth experiments with the well-studied strain PAO1, which has a relatively large QS regulon, under conditions where only limited QS-controlled functions are required. We grew P. aeruginosa for about 1000 generations in a condition where expression of QS-activated genes was required, and emergence of QS mutants was constrained and compared the QS regulons of populations after 35 generations to those after about 1000 generations in two independent lineages by using quorum quenching and RNA-seq technology. In one lineage the number of QS-activated genes identified was reduced by over 60% and in the other by about 30% in 1000-generation populations compared to 35-generation populations. Our results provide insight about the variations in the number of QS-activated genes reported for different P. aeruginosa environmental and clinical isolates and, about how environmental conditions might influence social evolution.

## INTRODUCTION

The opportunistic human pathogen Pseudomonas aeruginosa activates expression of a battery of genes by using acyl-homoserine lactone (AHL) quorum sensing (QS). There are two P. aeruginosa AHL QS circuits, the LasR-I and RhlR-I circuits. Genomic sequencing shows the genes for these circuits are highly conserved among hundreds of P. aeruginosa isolates from different environments (1). The *lasI* gene codes for a *N*-3-oxo-dodecanoyl-homoserine lactone (3OC12-HSL) synthase, and *lasR* codes for a 3OC12-HSL-dependent transcription factor, which activates dozens of genes. The *rhlI* gene codes for a *N*-butanoyl-homoserine lactone (C4-HSL) synthase, and the product of *rhlR* is a C4-HSL-responsive transcription factor, which activates a set of genes overlapping those activated by LasR (2). In strain PAO1, the RhlR-I circuit requires activation by the LasR-I circuit (3, 4).

Strain PAO1 has served as a model organism for studies of P. aeruginosa QS, studies of the involvement of QS in P. aeruginosa virulence, and studies of P. aeruginosa control of cooperative activities by QS. In fact, P. aeruginosa PAO1 has been used as a model to better understand the biology of AHL QS in general (2, 5). By comparing QS mutants to their parent, we have learned that well over 100 genes are activated by QS in strain PAO1 (6–8). Less is known about QS-dependent genes in other P. aeruginosa isolates, but limited transcriptomics studies have revealed that there is a core set of about 42 QS-activated genes common among several different strains isolated from different environmental and infection habitats. The size of the regulon in a given isolate can vary from a few dozen to upwards of 300 genes (6).

Transcriptomics, primarily of strain PAO1, revealed that many QS-activated genes code for extracellular proteins or production of extracellular products (7). This is consistent with the idea that QS serves as a cell-cell communication system, which coordinates production of public goods, extracellular factors that can be shared by all individuals in a group. Because QS controls public goods, these goods are only produced when population density is sufficient for them to provide a benefit (9, 10). This public goods hypothesis has been tested in several ways. For example, a prediction of the hypothesis is that, because there is a cost to cooperation, the public goods production cost, QS mutants should have a fitness advantage when competing with their parent. Growth of P. aeruginosa on proteins relies on QS activation of genes coding for extracellular proteases, and LasR mutants have a fitness advantage over their parent during co-culture on proteins like casein (9, 10). Furthermore, when P. aeruginosa PAO1 is transferred daily in a minimal medium with casein as the sole source of carbon and energy, LasR mutants emerge and reach a substantial fraction of the population within 10–20 days (10, 11). This is one evolutionary trajectory for the QS regulon: LasR mutant cheats can emerge in cooperating populations and either exist as a stable subset of the population or cause a collapse of a population if there are insufficient numbers of protease-producing individuals. Many previous descriptions of QS evolution in P. aeruginosa have focused on inactivating mutations of *lasR*. Such mutations are common in several environments, particularly chronic infections (12–17). In chronic infections, there is a selection against LasR QS, with the consequence of inactivating or significantly narrowing AHL QS in these bacteria (15, 18). Environmental bottlenecks can eliminate such mutants (19), and this can explain why LasR QS is conserved in terms of evolution. But why does the size and the scope of the QS regulon show strain-to-strain variability? This is the question we sought to begin to address.

As discussed above, when P. aeruginosa PAO1 is grown on casein, LasR QS mutants emerge as social cheats, which are cells that benefit from LasR-dependent extracellular protease production without producing the protease themselves (10). This complicates long-term experimental evolution experiments to address the question of why QS competent isolates might have different QS regulons. To execute long-term evolution experiments where LasR mutants are constrained we capitalized on a previous discovery that when strain PAO1 is provided with casein, for which its growth requires LasR-dependent production of secreted proteases, and adenosine for which its growth requires a cell-associated nucleoside hydrolase, LasR mutants did not emerge over the 30-day course of the study (11). Of course, growth of strain PAO1 on casein or casein plus adenosine are both contrived laboratory conditions and do not approximate the complex and variable conditions P. aeruginosa is exposed to in natural habitats be they environmental or human host associated. Nevertheless, the experiments we describe here provide a framework for understanding naturally occurring strain-to-strain variations in the composition of the P. aeruginosa QS regulon.

## RESULTS

### Long-term growth experiments.

**FIG 1:**
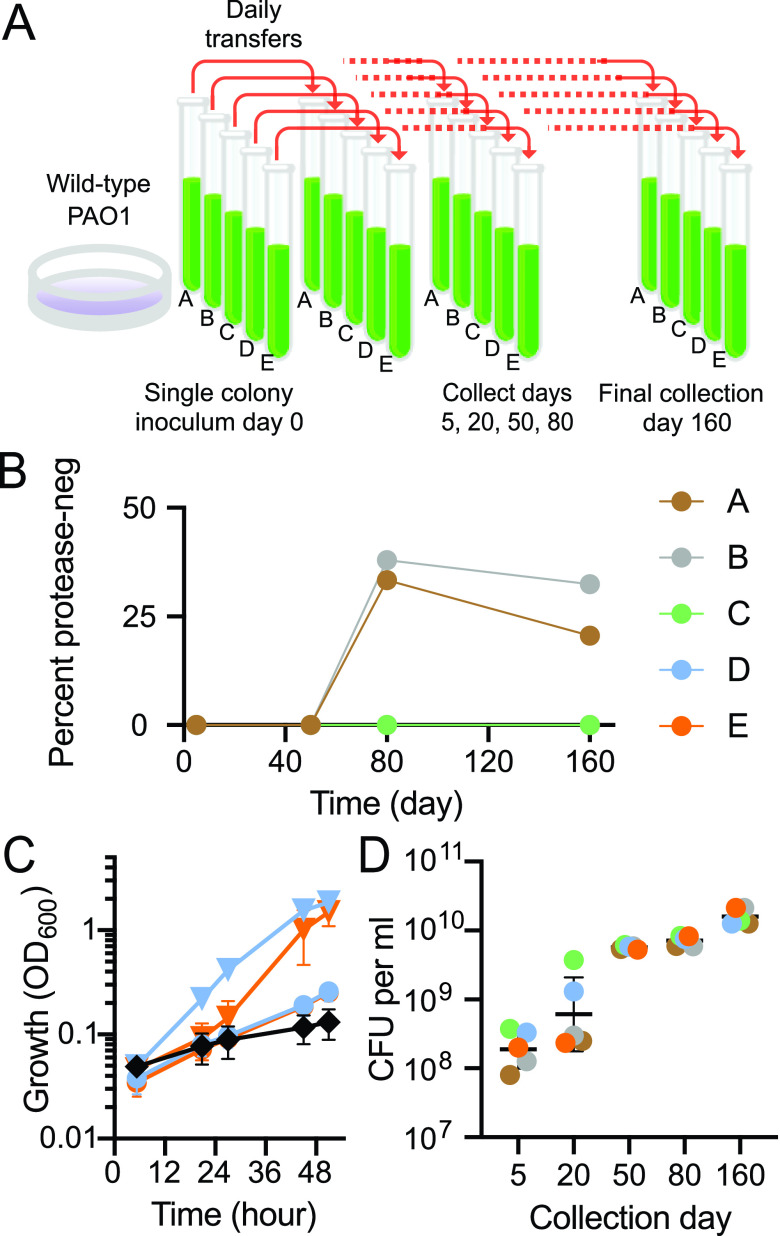
Long-term evolution of P. aeruginosa PAO1 serially passaged in a medium (CAB) that requires quorum sensing for growth. (A) Experimental design. (B) Abundance of protease-negative (neg) cheaters in each lineage (A through E) at the indicated days. (C) Growth of lineages D (blue) or E (orange) in adenosine-only broth. Inocula were cells from 5-day (circles) or 50-day (triangles) CAB cultures. Growth of the parent strain PAO1 (black diamonds) is included for comparison. Data are the means of two biological replicates, error bars are the range. (D) Growth yields (colony-forming units [CFU] per mL) of indicated populations grown in CAB for 18 h. Black lines are the geometric mean of three or four biological replicates for each population; error bars are the geometric standard deviation.

P. aeruginosa cells grown in buffered Lysogeny Broth (LB) were used to inoculate five tubes of a minimal broth containing only casein and adenosine (CAB) as carbon and energy sources. After inoculation, cells were transferred to fresh CAB daily for the next 160 days (Fig. 1A). A previous publication has shown that after the first several days of growth on casein, a PsdR mutant sweeps through the population. Mutations in *psdR* enhance growth on casein (20). The *psdR* gene codes for a repressor of a peptide transporter, and enhanced growth can be explained, at least in part, by improved uptake of peptide products of LasR-induced extracellular proteases. Growth on adenosine (or casein and adenosine) results in emergence of cells, which show improved growth on adenosine resulting from a genome amplification of a region containing the LasR-dependent *nuh* gene (21).

Protease-negative isolates from growth in casein are almost always LasR mutants (10). We screened populations from 5-day, 50-day, 80-day and 160-day cultures of all five lineages for protease-negative individuals. We did not observe any protease-negatives among the 100 individuals from each of the 5-day or 50-day cultures, but protease-negatives were identified in two of the 80- and 160-day populations (Fig. 1B). One likely explanation for breakthrough of protease-negative mutants by day 80 is that there is a mutation uncoupling adenosine metabolism from the QS-regulon such that LasR mutants do not incur a cost in CAB; they have a fitness advantage identical to that observed during growth of P. aeruginosa on casein alone.

We selected two of the three lineages (lineages D and E), in which LasR mutants were not detected because examination of these lineages allowed us to address the question of how QS regulons might differ in size and composition in isolation from the complication of a QS mutant cheater subpopulation. Early in the experiment at day 5, *psdR* mutations were not detected in either lineage D or E. At day 50 specific *psdR* mutations were fixed in each population. There was a frameshift mutation in lineage D (insertion of a C at position 431), and a T515C single-nucleotide polymorphism (SNP) in lineage E. This finding was consistent with the idea that the populations were adapting to growth on casein. We asked if there was also adaptation to growth on adenosine. When transferred to a minimal medium containing only adenosine as a carbon and energy source, day-5 populations of both lineages grew slowly, but day-50 populations grew more rapidly (Fig. 1C), as would be expected of a population with a *nuh* region amplification. A next question is how does evolution in CAB affect population growth? Because partial degradation of casein results in a milky haze in CAB, growth cannot be monitored by following optical density at 600 nm (OD). Therefore, we assessed growth yields by using plate counting to determine cell yields after 18 h in CAB for populations isolated after 5, 20, 50, 80 and 160 days, and found that there was a large increase in yield at day 50 (Fig. 1D). This corresponded to a time after *psdR* mutations had swept through the two populations, and the populations had acquired the ability to grow rapidly on adenosine (Fig. 1C). There was a further increase in growth yield in the day 160 populations (Fig. 1D). Because cultures were diluted into fresh CAB daily as a 1% inoculum there were six to seven doublings per day, corresponding to about 1,000 generations over the 160 daily transfers.

### Individual isolates from day-160 CAB cultures show diversity.

That day-160 CAB populations attained substantially higher growth yields than day-5 populations (Fig. 1D) likely resulting from an accumulation of multiple mutations. To assess the issue of mutation accumulation we sequenced the genomes of two isolates from lineage D and two isolates from lineage E day-160 CAB cultures. With the parent strain PAO1 as a reference we found between 148 and 167 SNPs in a given isolate (Table S1). We also identified large deletions or gene-duplication-amplification events at only two loci in both lineages. Both isolates from lineage E had a 6-kb deletion encompassing *pvdD* and *pvdJ*, genes required for synthesis of the iron siderophore pyoverdine (Table 1). Neither isolate from lineage D had this deletion; however, both D isolates possessed an internal gene duplication that would render the pyoverdine peptidase synthase, PvdI, inactive (Table 1). The lineage E isolates had an amplification of a genomic region of about 150 kb that included *nuh* (two copies in one isolate and three in the other). One of the two lineage D isolates had two copies of a 90-kb *nuh*-containing region and the other isolate did not have amplification of a *nuh*-containing region (Table 1). As discussed above, mutations in *psdR* have been reported to sweep through populations grown on casein alone after just a few daily transfers and in fact we found *psdR* mutations in all four of the day-160 isolates. As expected, the *psdR* mutations were the same as those fixed in the day-50 populations (Table 1). Including the *psdR* mutations, there were nonsynonymous or indel mutations in only 12 genes in all four of the sequenced isolates (Table 1, bold entries). We note that many of the mutations that we identified frequently occur in chronic infections and in experimental evolution experiments, such as those encoding efflux pump and pilus assembly genes (Table S1) (22–24). We also analyzed our RNA-seq data to identify nonfixed mutations that might be frequent in the populations. We did not find any with a population frequency greater than 15%, beyond those listed in Table S1.

**TABLE 1:** Genes with mutations in day-160 isolates^*a*^

Locus^*b*^	Mutation	160-day isolate	DNA modification^*c*^	Gene(s)^*d*^	Gene description^*d*^
Multiple	Gene duplication	D1	90.1 kb duplication (2 copies)^*e*^	PAO109-PAO192	Contains two genes (encoding a purine nucleosidase and an adenosine deaminase) that when duplicated confer fast growth on adenosine (Toussaint, et al. 2017)
		E1	154.7 kb duplication (2 copies)	PAO117-PA0260	
		E2	153.2 kb duplication (3 copies)	PAO122-PA0260	
**PA0143**	Intergenic SNP	D1, D2, E1, E2	C163308T	*nuh*	Purine nucleosidase Nuh
**PA0928**	Nonsynonymous SNP	D1, D2, E1, E2	C1420T	*gacS*	Histidine kinase sensor-response regulator
**PA1949**	Nonsynonymous SNP	D1, D2, E1, E2	C32T	*rbsR*	Ribose operon repressor RbsR
PA2338	Nonsynonymous SNP	E2	A635G		Probable binding protein component of ABC maltose/mannitol transporter
	Insertion	D1, D2	G856GC		
PA2399-PA2400	Deletion	E1, E2	6.2 kb deletion spanning 2 genes	*pvdD, pvdJ*	Pyoverdine synthetase D, J
PA2402	(Internal) gene duplication	D1	Internal 7.7 kb duplication (2 copies)	*pvdI*	Pyoverdine peptide synthetase I
		D2	Internal 6.9 kb duplication (4 copies)		
**PA2492**	Nonsynonymous SNP	D1, D2, E1, E2	T887C	*mexT*	Transcriptional regulator MexT
**PA2494**	Nonsynonymous SNP	D1, D2, E1, E2	G2069A	*mexF*	Multidrug efflux transporter MexF
PA2602	Insertion	D2	C338CG		3-mercaptopropionate dioxygenase
		E1, E2	C338CGG		
PA3205^*f*^	Insertion	E1	TG81TCC		Hypothetical protein
	Insertion	D2	TG81TGC		
**PA3488** ^*g*^	Deletion	D1, D2	AGG73A	*tli5*	T6SS-associated effector immunity protein
	Insertion	E1, E2	C280CA		
PA3527^*h*^	Intergenic insertion	E1, E2	G3947041GC	*pyrC*	Dihydroorotase
	Nonsynonymous SNP	D2	T527C		
**PA3535**	Stop gained	D1, D2, E1, E2	C2491T	*eprS*	Probable serine protease
**PA3548**	Intergenic SNP	D1, D2, E1, E2	G3974147A	*algI*	Alginate biosynthesis protein
**PA3620**	Deletion	D1, D2, E1, E2	TGC2067T	*mutS*	DNA mismatch repair protein MutS
PA3626	Nonsynonymous SNP	E1, E2	T232C	*ygbO*	tRNA pseudouridine synthase D
		D1	T658C		
PA3629	Nonsynonymous SNP	E1	T800C	*adhC*	Alcohol dehydrogenase
		D2	T1033C		
PA4344	Nonsynonymous SNP	E1, E2	G449C		Probable hydrolase
		D1	T953C		
**PA4499**	Insertion	D1, D2	A431AC	*psdR*	Dipeptide regulator
	Nonsynonymous SNP	E1, E2	T515C		
**PA4547**	Nonsynonymous SNP	D1, D2, E1, E2	T971C	*pilR*	Two-component response regulator PilR
PA4554	Deletion	E1, E2	GC341G	*pilY1*	Type IV pilus assembly protein PilY1
	Stop gained	D1	G962A		
**PA4701**	Nonsynonymous SNP	E1, E2	T26C		Conserved hypothetical protein
	Insertion	D1, D2	A44AC		
PA5090	Insertion	E1	G1277GC	*vgrG5*	T6SS-associated protein VgrG5
	Deletion	D2	GC1277G		

a For inclusion in this table there had to be a mutation in at least one of the two isolates from each lineage.

b Bold text indicates loci with SNPs or indels shared among all four isolates from the day-160 lineages with minimum read frequencies of 90% (except as in footnotes e-g).

c Nucleotide change and location within the coding sequence of the gene, unless the SNP is intergenic, which is identified by genome coordinate.

d As described at pseudomonas.com (47).

e Size of large structural variations (insertions or deletions larger than 100 bp) and copy number of duplicated genes.

f PA3205 SNP resulting in P31 frameshift was present in E1 with a frequency of 83% and in D1 supported by 71% variant reads.

g PA3488 SNP resulting in G27 frameshift was present in both isolates from lineage D supported by 97 and 85% variant reads, while the SNP resulting in H28 frameshift was present in lineage E with 89 and 96% variant reads.

h PA3527 SNP resulting in I176T amino acid change was supported by 100% variant reads in D2, and the intergenic insertion was present at 81 and 80% in lineage E.

10.1128/mbio.00161-22.1 TABLE S1 Mutations in day-160 isolates from lineages D and E. Download Table S1, PDF file, 0.2 MB. Copyright © 2022 Smalley et al. 2022 Smalley et al. https://creativecommons.org/licenses/by/4.0/ This content is distributed under the terms of the Creative Commons Attribution 4.0 International license.

### Validation of a population transcriptomics approach.

Valuable information about the P. aeruginosa QS regulon has been gained by transcriptome analysis using QS gene deletion mutants (6–8), but transcriptomics is limited by the time point chosen for identification of QS-activated genes. Many genes in the P. aeruginosa QS regulon are coregulated by other factors (25). Some genes show delays in QS activation compared to other genes (7). Because we sought to identify changes in the cohort of genes activated by QS between day 5 and day 160 of our experiment, we performed a transcriptome comparison of *populations* with active QS against *populations* with QS inhibited. For this analysis we had to overcome two challenges. First, our transcriptomics experiment could not be performed in CAB. Because QS is required for CAB growth, QS inhibited populations cannot grow on CAB. Therefore, we assessed composition of the QS regulon of cells grown in LB. Besides the fact that this is possible, it also allows for comparisons to most previous transcriptome analyses of the QS regulon as the majority have been with LB-grown cells, and this more directly addresses the question of how the regulon has been altered generally. Second, previous transcriptome investigations have compared wild-type bacteria to QS mutants. However, our genome sequencing and our population SNP analysis showed that population diversity developed during our 160-day experiment. Therefore, isolating one or even several individuals from the population, generating QS mutants, and comparing them to their parents would not be representative of the population behavior. Therefore, we determined QS regulons in populations rather than individual isolates. For this metatranscriptomics approach we grew populations in LB plus or minus an AHL-degrading lactonase, AiiA (26), used by others to identify QS-activated genes in pure cultures of bacteria (27).

Because many of the genes in the P. aeruginosa QS regulon are coregulated by other factors (25), they cannot all be identified by transcriptomics of cells taken from any specific point in growth, but previous investigations have shown that most can be identified at a culture optical density at 600 nm (OD) of 2 in LB (6, 7). Thus, our analyses were with RNA harvested from cells grown to this OD. We note that, unlike CAB (Fig. 1D), where the 160-day populations reached a higher cell density than day 5 cultures, growth of the 5-day and 160-day populations was indistinguishable in LB (Fig. S1). Addition of AiiA lactonase did not affect the LB growth of any of the P. aeruginosa populations and reduced AHLs to below detectable levels (<0.001 μM and <0.005 μM for 3OC12-HSL and C4-HSL, respectively). We determined levels of 3OC12-HSL and C4-HSL in LB culture fluids of lineages D and E of 5-day and 160-day populations at an OD of 2.0. In all cultures without AiiA, regardless of lineage or days of CAB passage, we found similarly high levels of 3OC12-HSL (range, 1.6 to 3.3 μM). C4-HSL levels were similar in both day-5 lineages (range, 1.6 to 4.4 μM); however, day-160 populations produced less C4-HSL (population D, 0.12 – 0.14 μM, population E 0.68–0.84 μM) than did Day-5 populations. To eliminate the possibility that variability in signal production might affect results of the transcriptomics experiments we added AHLs (2 μM 3OC12-HSL and 10 μM C4-HSL) to cultures of populations grown without AiiA lactonase. We note that these additions are similar to those used by others (6, 7) to study QS regulons of AHL synthesis mutants.

10.1128/mbio.00161-22.6 FIG S1 Growth of 5-day and 160-day populations in LB. Preparation of inocula and growth conditions were as described in the RNA-Seq Analyses section of the Materials and Methods. The data are means of two biological replicates and error bars indicate the ranges. Most error bars are smaller than the symbols. Download FIG S1, PDF file, 0.03 MB. Copyright © 2022 Smalley et al. 2022 Smalley et al. https://creativecommons.org/licenses/by/4.0/ This content is distributed under the terms of the Creative Commons Attribution 4.0 International license.

We first compared the transcriptomes of lineage D and E populations taken from the fifth day of passage on CAB to each other. Transcripts showing >2.8-fold (1.5-fold, log_2_) higher levels in populations grown with added AHLs compared to populations grown with added AiiA lactonase were considered QS activated. This fold change cutoff was selected as it captured a previously determined core QS regulon (6) and limited the false discovery rate to <0.05. We identified 193 QS-activated genes in lineage D and 148 in lineage E (Table 2). There were 137 genes common to both lineages. These differences between lineages D and E may reflect a combination of independent evolutionary trajectories and the vagaries of our transcriptome analysis. There is reason to believe that it may be the latter: most of the QS-activated genes identified in one, but not the other, lineage were minimally above the 2.8-fold threshold for classification as QS activated (Table 2). Several of these genes were in operons where other genes exceeded the cutoff in both lineages. For example, 11 genes of the *hcp* secretion island III (HSI-3) cluster of type VI secretion system genes (PA2361-PA2371) in lineage D were classified as QS-activated, whereas only four of these genes in lineage E were classified as activated. Of the 137 QS-activated genes common to both lineages (Table 2), 126 (>90%) have previously been identified as such (6, 7). For the 42 genes defined as core QS-activated genes (6), 41 were QS-activated in both lineages D and E in the day-5 populations (Table 2). These results indicated that this transcriptomics approach would suffice to address our primary questions: does long-term growth in CAB lead to a reduction in the size of the QS regulon, and if so, what genes are eliminated from the regulon?

**TABLE 2:**
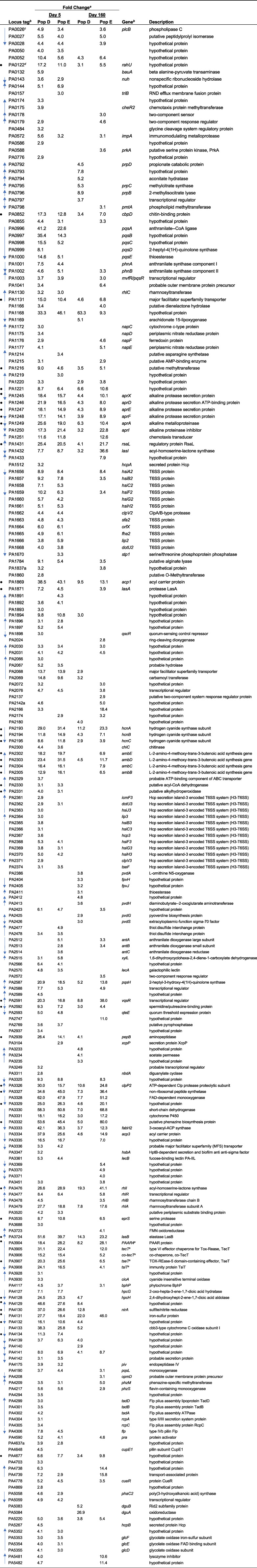
Quorum sensing activated genes in day-5 and day-160 CAB populations

a Differential gene expression of populations grown with added AHLs vs. added AiiA.

b Locus tag and gene name from pseudomonas.com (47) except where indicated.

c Arrows adjacent to loci indicate operons and their direction of transcription as described in reference 48.

d Closed circles indicate that the gene was identified as part of the core quorum sensing regulon defined in reference 6.

e Gene names as described in reference 49.

### Populations show a reduction in the QS regulon after 160 days in CAB.

**FIG 2:**
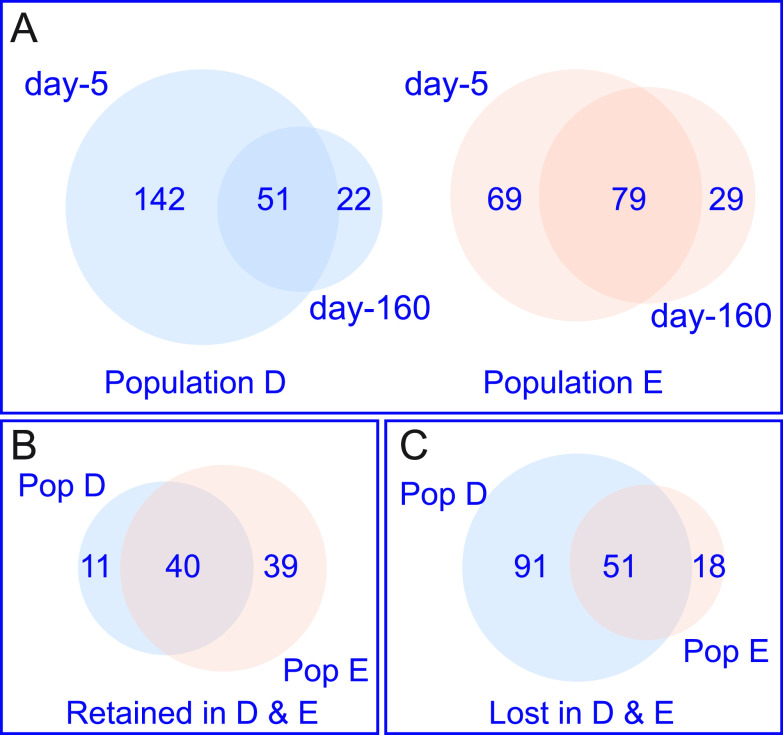
Many QS-activated genes in day-5 populations do not show QS activation in day-160 populations. (A) Venn diagrams showing the relationship between QS-activated genes at day-5 versus day-160 populations for lineages D (blue) and E (peach). (B) Venn diagram showing the overlap of genes that remain under QS-control (Retained) in both populations after 160 days of serial passage in CAB. (C) Venn diagram showing the overlap of genes that are no longer under QS-control (Lost) in both populations. Numbers in the Venn diagrams were determined using Venny (46) and area calculated using the area-proportional Venn diagram plotter and editor found at http://apps.bioinforx.com/bxaf7c/app/venn/index.php. The numbers of genes in each category are indicated. Lists of genes shared in each category are in Table 2.

We compared the QS-activated genes in day-160 populations to those in the respective day-5 populations (Table 2) and found the number of QS-activated genes in lineage D was reduced from 193 at day 5 to only 73 at day 160 (Fig. 2A). For lineage E the number went from 148 to 108. There were a few genes that showed QS-activation in one, the other, or both day-160 populations but not in the day-5 populations.

**FIG 3:**
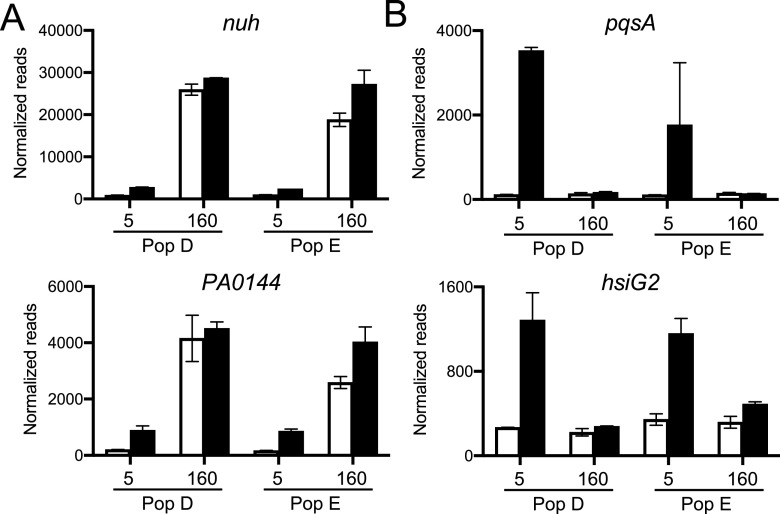
Normalized RNA-seq reads of select genes that are lost from QS control during serial passage in CAB for 160 days. (A) The only two lost genes with increased expression, *nuh* and PA0144, after 160 days of CAB passage. (B) Two lost genes, *pqsA* and *hsiG2*, with low expression levels in 160-day populations even in the presence of added AHLs. White bars are AiiA-treated and black bars are plus AHL signals. Data are the mean normalized transcript counts of the two biological replicates for populations (D or E) passaged for 5 or 160 days in CAB; error bars represent the range.

The loss of QS-gene activation after long-term growth in CAB could indicate a gene is expressed at levels similar to or greater than the QS-activated levels early in growth on CAB, or it could indicate a suppression of gene expression to levels similar to those in early populations grown in the presence of AiiA lactonase. To address this issue, we examined expression of the 51 genes that showed QS-activation in both early populations, but not in both late populations. Only two of the 51 genes showed high levels of expression in day-160 populations, *nuh* and the adjacent gene PA0144 (Fig. 3A). The high expression of *nuh* and PA0144 was expected from previous studies (21). The other 49 genes were expressed at very low levels in both of the 160-day populations in the presence of added AHLs (Fig. 3B). This finding is consistent with the idea that the decreased size of the QS regulon after 160 days of growth on CAB is economical.

It is possible that the observed loss of genes from the QS regulon could reflect a more global pattern of gene expression, not specific to the QS regulon. That perhaps there was a general lowering of the expression of genes not relevant to growth in CAB. To address this possibility, we compared gene expression in populations grown with AHLs from day 160 versus day 5. Of the 5570 PAO1 genes (28), we found that 379 genes in population D and 362 in population E, with 186 in common, were expressed at low levels in LB cultures of day-160 compared to day-5 populations (Table S2). About 20% of the 186 common genes in this analysis were in the QS regulons. These data indicate that downregulation of gene expression in our experiment was concentrated on the QS regulon. We also found 177 genes that were more highly expressed in both populations at day 160 than at day 5 (Table S3). Not surprisingly, many of these genes encode products involved in catabolism of adenosine (such as *nuh*) or of the constituents of casein (such as a dipeptide transporter, PA4500-4506).

10.1128/mbio.00161-22.2 TABLE S2 Genes with decreased expression at day 160 in CAB-evolved Populations D and E, compared to day 5. Download Table S2, PDF file, 0.1 MB. Copyright © 2022 Smalley et al. 2022 Smalley et al. https://creativecommons.org/licenses/by/4.0/ This content is distributed under the terms of the Creative Commons Attribution 4.0 International license.

10.1128/mbio.00161-22.3 TABLE S3 Genes with greater expression at day 160 in CAB-evolved Populations D and E, compared to day 5. Download Table S3, PDF file, 0.1 MB. Copyright © 2022 Smalley et al. 2022 Smalley et al. https://creativecommons.org/licenses/by/4.0/ This content is distributed under the terms of the Creative Commons Attribution 4.0 International license.

### Characteristics of genes retained in the QS regulon after 160 days of growth in CAB.

As expected, the genes for AHL synthesis, *lasI* and *rhlI*, are retained in the day-160 QS regulons of both lineages (40 genes in common are retained, Fig. 2B). Genes for production of extracellular proteases are also retained, including the elastase gene, *lasB*, and the alkaline protease, *apr* genes (Table 2). Of note, not all genes coding for an extracellular protease are maintained in the regulon. For example, *piv*, which codes for an endopeptidase is no longer QS-controlled in either day-160 populations. Not all the genes retained in the QS regulon are obviously related to adenosine or casein digestion. For example, *hcnABC*, encoding a hydrogen cyanide synthase, remains QS-regulated; hydrogen cyanide has previously been shown to be important in policing of QS mutants (29, 30). For other gene products, the relation to growth in CAB is unclear. It may be that they are retained in the regulon because they serve some purpose during growth on CAB. The significance of this finding is yet to be determined.

### Characteristics of genes lost from the QS regulon after 160 days of growth in CAB.

There were 51 genes lost from the QS regulons of both populations D and E (Fig. 2C). There was a loss of genes related to Type VI secretion or Type VI-secreted products from the QS-regulons of both lineages. For example, 9 of the 12 genes encoded by the Hcp secretion island II (HSI-2) cluster of T6SS genes (PA1656-PA1668) were eliminated from the QS regulons of lineages D and E by day 160, as were other T6SS-related genes throughout the genome. Three genes considered core QS-activated genes (6) were not activated in the day-160 populations of either lineage. All three (PA2330, PA2331, and PA4134) encode hypothetical proteins and showed substantial activation in day-5 populations but did not reach our threshold for activation in day-160 populations. Other genes lost from the regulon include the chitinase gene (*chiC*) and two lectin genes (*lecA* and *lecB*), that may provide benefits in myriad environments, but not in CAB.

### Diverse routes to elimination of a gene from the QS regulon.

The elimination of a given gene from the QS regulon by day 160 in CAB could have many possible explanations. For example, there can be a mutation in the promoter for that gene, there can be a delay in the QS activation of that gene such that it falls below the cutoff for activation at an OD of 2, there could be a mutation in a co-activator of that gene, or there could even be a mutation in that gene. Some of these possibilities have long-term consequences, and others can perhaps be more readily reversed if conditions change.

**FIG 4:**
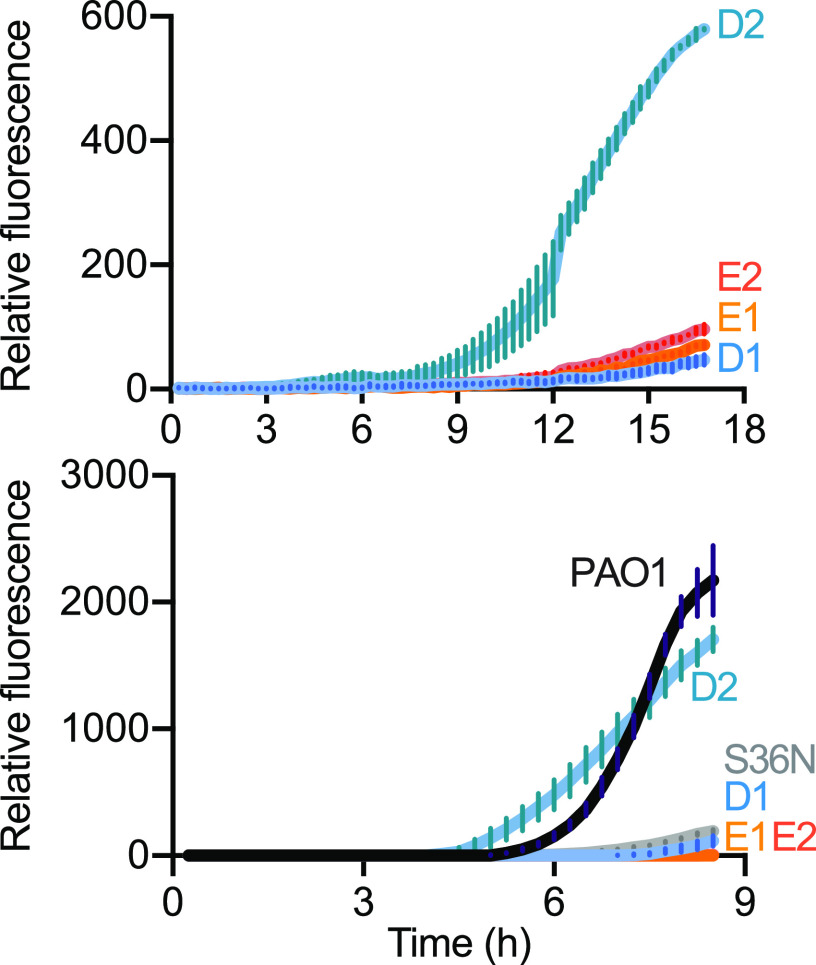
There are diverse routes to eliminate *pqsA* from the QS regulon. GFP fluorescence in isolates containing a P*_pqsA_-gfp* reporter plasmid, grown in 96-well plates in CAB (top) and LB (bottom). The strains include isolates evolved for 160 days in CAB for population D (D1, D2) or E (E1, E2), the parent strain (PAO1), and a PqsR-variant mutant strain (S36N). Data are the mean relative fluorescence units over time of three technical replicates for two biological replicates; error bars represent standard deviations of means.

To begin to address the question of how a gene identified as QS activated in day-5 populations might not be QS activated in day-160 populations, we examined promoter activity of the QS-regulated gene *pqsA* in our four day-160 clones used for genome sequencing (two each, lineages D and E). We selected *pqsA* because there was no mutation in its promoter region in either lineage, but there were variants in *pqsR* (also called *mvfR*), the LasR-regulated gene encoding the transcriptional activator of *pqsA* (Table S1). We transformed the four isolates with a *pqsA-gfp* reporter plasmid and followed GFP fluorescence during growth in CAB. The two day-160 population E clones showed low, basal expression of the *pqsA* reporter over 16 h of growth, indicating a loss of cell density-dependent expression associated with QS (Fig. 4A). In contrast, only one of the two population D clones showed a similar loss in density-dependent *pqsA* expression, while the other showed *pqsA* expression consistent with QS activation.

Our genomic sequence analyses identified an identical SNP in *pqsR* in both lineage E clones. The SNP codes for a S36N substitution. We reasoned that the PqsR S36N mutation could explain the loss of *pqsA* activation by QS in our lineage E day-160 clones. To determine whether the PqsR S36N mutant is inactive and unable to activate expression of *pqsA*, we constructed a PAO1 *pqsR* S36N mutant and transformed both the mutant and PAO1 with the *pqsA-gfp* reporter. We monitored GFP-fluorescence over a growth curve, but this time in LB because the PAO1 wild type grows slowly on initial transfer to CAB. The S36N PAO1 mutant showed minimal GFP fluorescence compared to PAO1, similar to the lineage E clones (Fig. 4B). For the sake of completeness, we also monitored GFP expression from the *pqsA-gfp* reporter in day-160 lineage D and E clones; the results were consistent with those from growth in CAB (Fig. 4A). We conclude that the PqsR S36N mutation explains the loss of *pqsA* from QS regulation in lineage E. The varied, but delayed, expression of *pqsA* in the isolates from population D do not have as obvious of an explanation. Clearly, there are different evolutionary trajectories leading to loss of or delayed expression of genes like *pqsA in* P. aeruginosa populations during prolonged CAB growth.

## DISCUSSION

We devised a strategy to determine whether QS-dependent growth of P. aeruginosa for about 1000 generations in one specific growth medium (CAB) would affect the constitution of the QS regulon, and our results show that the regulon was substantially reduced. Our studies on P. aeruginosa can be put in the context of the rich literature on laboratory evolution of Escherichia coli, *which* has been used as a model to investigate long-term evolution over a span of tens of thousands of generations in a specific growth environment. For E. coli subjected to daily transfers to a fresh medium containing a single carbon and energy source, population fitness gains occur over time and certain mutations can sweep through a population (31). The sweeps result in a gain in fitness. Fitness improvements are most dramatic early and become progressively smaller over time and generations. Multiple variants coexist in each population and evolutionary trajectories are complicated and varied (32). Although our study was focused on the QS circuitry of P. aeruginosa, it reflected many of the findings from E. coli, including the timing of fitness gains (Fig. 1D) and multiple evolutionary trajectories (Fig. 1B).

It is notable that, unlike E. coli, our experimental subject, P. aeruginosa, is notorious for undergoing genetic changes during routine laboratory maintenance. In fact, strain PAO1 cultures that have been maintained in different laboratories show significant different genotypic and phenotypic characteristics (33). Compared to E. coli, much less is known about evolution of P. aeruginosa during adaptation to a constant environment. There is some information about adaptation to environments where P. aeruginosa QS is required for growth (2). In a relatively short time during QS-dependent growth on casein, a mutation in *psdR*, which codes for a repressor of a small-peptide uptake system, sweeps through the population and results in a substantial fitness gain reflected by more rapid growth in this environment (20). Inactivating mutations in the transcription factor *mexT* or the *mexEF-oprN* efflux pump genes also improve growth on casein, at least in part, by increasing activity of the Rhl QS system (34, 35). During QS-dependent growth on adenosine, a poor energy source for P. aeruginosa PAO1, amplification of a genomic region containing the adenosine hydrolase gene *nuh* arises and results in a substantial growth improvement (21). Growth on casein requires the QS-induced public extracellular protease elastase (LasB), and after *psdR* sweeps through the population, LasR QS mutants emerge. The LasR mutants, which do not activate any of the many genes in the QS regulon, have a negative-frequency fitness advantage over their parents during growth on casein (2, 9, 10). QS-dependent growth on adenosine requires the periplasmic *nuh* product, purine hydrolase, a private good. In short-term evolution experiments (30 days, about 200 generations) on casein and adenosine together, QS mutants are constrained. Our interpretation of this constraint is that LasR mutants can benefit from proteases produced by their parents, but they have lost access to adenosine (11).

In any given environment QS may be important for fitness as a result of activation of only a small number of genes. A fitness cost would be incurred by expression of the many other QS-activated genes. One solution to this fitness problem might be a reduction in QS activation of a large fraction of the QS regulon. There are several issues related to such a possibility. How might this be facilitated mechanistically? Might such a reduction in any given isolate reflect its ecological history? Would this provide a possible benefit in that perhaps a silenced gene could be returned to the QS regulon if ecological conditions are altered? The gene pool can remain intact when fitness is a result of rewiring gene regulation. There is circumstantial evidence to support the idea that the QS regulon might reflect the ecological history of a given isolate. In a limited comparison of QS-activated genes in seven isolates from different habitats, Chugani and colleagues found diversity in the regulons of QS-activated genes among various isolates (6).

To facilitate our experiments, we transferred populations daily in a medium containing both casein and adenosine (CAB) as the sources of carbon and energy. Protease-negative mutants were constrained in all five of the lineages we maintained for at least 50 days. By day 80, protease-negative mutants had broken through in two of the five lineages (Fig. 1B). We speculate that in these two lineages, the restraint on cheating (11) provided by QS induction of a cellular product, Nuh, was relieved through mutations in the *nuh* promoter that increase its transcription, even in LasR-null individuals (21). These results are consistent with the idea that in a particular environmental condition, pleiotropy may be insufficient to maintain cooperation in populations (36) and breakthrough variants may occur. However, metabolic constraints might be an effective deterrent to cheating in real-world environments where available resources are likely to be more complex and variable, and where population bottlenecks may occur.

We focused our investigations on two of the populations that continued to constrain LasR mutants at day 160. We did not pursue the LasR mutant breakthrough populations where analysis would certainly be more complicated. Similar to E. coli, fitness improvements were most dramatic early (with large increases in fitness between days 5, 20, and 50) and became progressively smaller over time (Fig. 1D). In fact, *psdR* mutations arose in both populations sometime between day 5 and day 50. It was also during this period that populations acquired the ability to grow rapidly on adenosine (Fig. 1C): there was both genotypic and phenotypic heterogeneity within each population. This heterogeneity suggests an interesting possibility, and it creates an experimental dilemma. The possibility is that there might be competition for resources among the individuals in the population. We have not yet addressed this possibility. The dilemma is that one cannot expect to learn about the breadth of the QS regulon by isolating a small number of individuals, creating QS mutants, and comparing transcriptomes of mutants to parents. To address this problem, we used a metatranscriptome method to interfere with population-level QS by using an AHL lactonase. Purified AiiA lactonase has been employed to study QS gene activation and phenotypes in bacterial pure cultures (27, 37–39). Here, we extend the use of an AHL lactonase to enable metatranscriptomics in heterogeneous populations. This approach might also be employed to study mixed-species populations.

Our QS metatranscriptomic analysis revealed marked reductions in the number of genes activated by QS in day-160 populations compared to day-5 populations. In one population (D) the number of QS activated genes at day 160 was only 26% of the number at day 5, and in the other population (E) it was 53%. One way to eliminate a gene from the QS regulon is by deletion, but our RNA-seq results and our genome sequencing of two isolates from each population indicated this was not a common event. There were only three deletions identified in the analysis, compared to hundreds of SNPs. It appeared that almost all QS regulon genes were intact. Their loss from the QS regulon could result from any of a several mechanisms, including delay in QS gene activation, modification of a coregulatory pathway, and subtle changes in levels of other transcriptional regulators in cells. Regardless of the mechanism, this seems to represent a flexible solution to reducing the cost of QS when P. aeruginosa populations thrive in an environment where only a few QS activated genes provide a benefit. Although yet untested, we hypothesize that modifying the environment in one way or another to involve QS activities other than protease or adenosine metabolism could further alter the regulon of bacteria from day-160 populations, and bring genes back under QS control.

To begin to understand ways in which P. aeruginosa reduced its QS regulon during long-term growth in CAB we focused on *pqsA*, a gene that showed greater than 10-fold QS activation in either day-5 population but not in either day-160 population (Table 2, Fig. 3B). *pqsA*, the first gene in the *pqs* operon, is required for production of the Pseudomonas Quinolone Signal (PQS). The *pqsA* promoter is activated by the PqsR transcription factor together with PQS, and *pqsR* transcription is activated by LasR. We found a point mutation in the two day-160 lineage E isolates, and this mutation was in fact fixed in the day-160 population. This presents a solution to the question of how the *pqs* operon is removed from QS activation and silent. In this solution, the pseudogene leaves an inactive copy of *pqsR*. It is conceivable that Pqs production can be recovered by repair of the pseudogene or expression of the *pqs* operon can become independent of PqsR. The two population D isolates showed different *pqsA* expression patterns in pure culture. One exhibited a low level of *pqsA* expression and the other showed delayed expression. Neither had a *pqsR* mutation. This population developed a different strategy, yet unknown, to release *pqsA* expression from QS activation. This finding is consistent with our other results indicating the populations are heterogeneous.

That genes can be lost from QS-control under the specific conditions of our experiment supports the view that assessing the QS regulon of a P. aeruginosa isolate might provide information about the ecological history of that isolate. We note that some of the differences between day-160 lineages and between day 5 and day 160 in a given lineage might be due to the noise inherent in RNA-seq analyses. Nevertheless, during our experiment, there was a reduction in the number of genes activated by QS. The increased population fitness observed between day 50 and day 160 in our metapopulations (Fig. 1D) may, in part, result from this economization in the size of the QS regulon.

## MATERIALS AND METHODS

### Bacterial strains, plasmids, and growth conditions.

Strains, plasmids, and primers used are listed in Tables S4 and S5. To create the PqsR S36N variant strain we used a homologous recombination-based two-step allelic exchange approach as described previously (35). Bacteria were grown in either lysogeny broth (modified to use 0.5% NaCl [40]) buffered with 50 mM 3-(N-morpholino) propanesulfonic acid at pH 7.0 (LB) or the minimal medium described in (41) with 0.25% caseinate and 0.75% adenosine (CAB) (11) or 1% adenosine-only. Bacterial growth was in 4 mL of broth at 37°C with shaking (250 rpm) in 18 mm tubes unless otherwise indicated, or in 200 μL volumes in 96-well plates where indicated. When required, gentamicin was added to the medium (10 μg/mL E. coli, 100 μg/mL P. aeruginosa). Screening for protease-negative colonies was on skim milk agar plates as described elsewhere (10). For colony isolation and plate counting we used LB plus 1.5% agar plates.

10.1128/mbio.00161-22.4 TABLE S4 Strains used in this study. Download Table S4, PDF file, 0.03 MB. Copyright © 2022 Smalley et al. 2022 Smalley et al. https://creativecommons.org/licenses/by/4.0/ This content is distributed under the terms of the Creative Commons Attribution 4.0 International license.

10.1128/mbio.00161-22.5 TABLE S5 Plasmids and primers used in this study. Download Table S5, PDF file, 0.03 MB. Copyright © 2022 Smalley et al. 2022 Smalley et al. https://creativecommons.org/licenses/by/4.0/ This content is distributed under the terms of the Creative Commons Attribution 4.0 International license.

### Long-term growth experiments and phenotypes.

Evolved populations were derived from wild-type P. aeruginosa strain PAO1 as diagramed in Fig. 1. Five individual colonies were used to inoculate 5 tubes of LB. From overnight LB cultures, 100 μl was used to inoculate 4 mL of CAB. After a 24-h incubation, 40 μl of culture (1% vol/vol) was transferred to 4 mL of fresh CAB. This process continued for 160 days. One-mL volumes of each population were collected at days 5, 20, 50, 80, and 160 and stored as glycerol (25% vol/vol) stocks at −80°C. To screen for protease-negative isolates we plated onto LB-agar and then patched 100 individual colonies onto skim milk agar plates (10). Colonies that lacked a zone of clearing on the milk plates were scored as protease negative. To look for *psdR* mutations, scrapings of day-5 and day-50 population glycerol stocks were used to inoculate LB. After overnight incubation, cells were pelleted by centrifugation and genomic DNA was extracted using a Gentra Puregene Yeast/Bacteria kit (Qiagen, Germantown, MD). The purified DNA was used as the template for PCR (*psdR* primers provided in Table S5) and the PCR product Sanger sequenced. For growth in 1% adenosine-only broth (Fig. 1C), scrapings of day-5 and day-50 population glycerol stocks or individual colonies of PAO1 were used to inoculate LB and incubated overnight. Overnight cultures were back-diluted in 1% adenosine-only broth to an OD of 0.01 and growth monitored by OD. To determine cell yields in CAB, scrapings of glycerol stocks were used to inoculate LB and incubated overnight. A 3-mL CAB culture was then inoculated with 30 mL of overnight culture, incubated at 37°C with shaking for 18 h, and then serially diluted for plating on LB agar to determine the number of CFU.

### Genome sequencing and variant analysis.

Scrapings of day-160 population glycerol stocks were streaked for isolation on LB agar plates. After an overnight incubation, two individual colonies from population D and two from population E were used to inoculate LB. After overnight growth, genomic DNA was extracted from cells and purified as described above. The purified DNA was used to construct paired-end 2 × 150 bp libraries for sequencing on an Illumina MiSeq (San Diego, CA). Reads were aligned to the PAO1 reference genome (accession NC_002516) using StrandNGS version 3.3.1 (Strand Life Sciences, Bangalore, India). Variant analysis was performed with StrandNGS SNP, CNV and SV pipelines. We defined a SNP as having a variant read frequency of >90% and an indel as being <100 bp. Previously, we sequenced the genomes of two individual isolates of our laboratory PAO1 strain, MPAO1 (SAMN066895, SAMN09671539), and we used this sequence for comparisons for SNPs calls and copy number/indel analyses.

### Purification of AiiA lactonase.

The AiiA AHL lactonase was purified as a maltose-binding protein (MalE) fusion from recombinant E. coli as described elsewhere (42) except that 0.2 mM CoCl_2_ was substituted for ZnCl_2_ and the MalE fragment was not removed. Purified AiiA was concentrated to 10 mg/mL in 20 mM Tris-HCl, 100 mM NaCl, pH 7.4 with 10% glycerol (vol/vol) and stored at −80°C until use. AiiA was added to cultures at a final concentration of 100 μg/mL, which was sufficient to reduce 3OC12- and C4-HSL levels to below detection as measured by bioassay (43).

### RNA-Seq analyses.

Scrapings from glycerol stocks of populations D and E (from day-5 and day-160 passages) were used to inoculate 3 mL of LB. After an overnight incubation, cells from these cultures were used to inoculate 3 mL of LB with a starting OD of 0.01. These cells were grown to logarithmic phase (OD 0.1–0.4) and then used to inoculate 25 mL of LB in a 250-mL baffled flask (starting OD 0.005) and one of the following was added: AiiA lactonase (100 μg/mL), AHLs (2 μM 3OC12-HSL and 10 μM C4-HSL final concentrations), or no additions. Two biological replicates were prepared for each condition. At an OD of 2.0, AHL signals were solvent extracted from 5 mL of culture and measured by bioassay as described previously (43). At the same time, cells from 2 mL of culture were pelleted by centrifugation, preserved in RNAprotect bacteria reagent (Qiagen, Germantown MD), and stored at −80°C, followed by RNA extraction as described previously (37).

RNA-Seq library preparation, bacterial Ribo-Zero rRNA depletion (Illumina, San Diego, CA), and sequencing was performed by Genewiz (South Plainfield, NJ). Library samples were divided into two separate HiSeq3000 runs, each with paired-end 2 × 150 bp read lengths. Trim Galore! (Babraham Bioinformatics, Cambridge UK) was used to trim adapters prior to alignment against the PAO1 reference genome (accession NC_002516) using StrandNGS version 3.3.1 (Strand Life Sciences, Bangalore, India). DESeq2 (44) was used for differential expression analysis, using the Benjamini-Hochberg adjustment for multiple comparisons and a false-discovery rate α = 0.05. Samples from each treatment regimen were grouped by individual population for the DESeq2 differential expression analyses (i.e., the three treatments for lineage D day-5 populations described above were compared separately from the day-160 population treatments, and likewise for lineage E populations). QS-activated regulons were determined by comparing added AHLs to AiiA-treated samples for a given population (D or E) and passaging time (5-day or 160-day) and imposing a 2.8-fold minimal fold change threshold (this is 1.5-fold on a log_2_ scale). Normalized transcript counts (Fig. 3) were obtained by taking the inverse log of the regularized log transformation (rlog) function in DESeq2 (44). Note that because there are two phenazine biosynthesis *phz* operons in the PAO1 genome with high levels of sequence identity, it is not possible to differentiate from which operon the transcripts derived, as the StrandNGS software assigns reads that match multiple locations (<5 matches) to the earliest match location in the genome. Thus, although phenazine synthesis is QS-regulated, we excluded *phzA1-G1* and *phzA2-G2* from subsequent analyses of our differential expression data.

### *pqsA-gfp* reporter experiments.

We created a promoter probe fusion for *pqsA* and a promoterless *gfp* control (Table S5) by using PCR and E. coli-mediated DNA assembly (45). Plasmids were used to electrotransform P. aeruginosa strains. Single transformant colonies harboring pBBR-P*_pqsA_*_-_*gfp* or pBBR-*gfp* were used to inoculate LB supplemented with gentamicin. Overnight cultures were back diluted to OD 0.01, grown to midlog phase (OD_600_ 0.1–0.4), diluted to an initial OD of 0.01 in CAB or LB as indicated, and dispensed in 200 μl volumes microtiter dish wells. Fluorescence (excitation 485 nm, emission 535 nm) and absorbance (OD_600_) was measured every 15 min for 14 h using a Biotek Synergy H1 microplate reader. Background fluorescence was determined using the promoterless pBBR-*gfp* control.

### Sequence data deposition.

Raw sequencing reads for variant analyses of the two individual isolates from each of the day-160 lineages (BioSamples SAMN16400702-SAMN16400711) were deposited in the NCBI Sequence Read Archive under BioProject PRJNA667949. Raw sequencing reads and count matrices for transcriptome analyses of CAB-evolved populations D (BioSamples SAMN16400698, SAMN16400699) and E (BioSamples SAMN16400700, SAMN16400701) were also deposited under GEO accession GSE176411.
