# Supplementary material for: Evolution of the Quorum Sensing Regulon in Cooperating Populations of Pseudomonas aeruginosa
Source: mBio. 2022 Feb 22;13(1):e00161-22. doi: 10.1128/mbio.00161-22 (PMC8863103; doi:10.1128/mbio.00161-22)
Supplement: TABLE S1 [file mbio.00161-22-st001.pdf]

Supplemental Table 1. Mutations in day-160 isolates from lineages D and E.

DNA Coordinates[a]					Population D		Population E								
Start	End	Reference	Variant Allele[b]	Variant Type	Isolate		Isolate		Locus Tag[c]	Gene Name[c]	Gene Description[c]	SNP Effect	Position in Gene[d]	Position in Protein[e]	Amino Acid Change
12262	12262	G	A	Substitution	D1	D2			PA0008	*glyS*	glycyl-tRNA synthetase beta chain	Non-synonymous	227-227	76-76	P->L
18253	18253	C	T	Substitution	D1	D2			PA0016	*trkA*	potassium uptake protein TrkA	Non-synonymous	487-487	163-163	G->S
27138	27138	C	T	Substitution	D1	D2			PA0025	*aroE*	shikimate dehydrogenase	Non-synonymous	428-428	143-143	P->L
42148	42148	G	C	Substitution			E1	E2	PA0040		putative hemolysin activation/secretion protein	Non-synonymous	1036-1036	346-346	V->L
43717	43719	GCG	GAA	Substitution			E1	E2	PA0041		probable hemagglutinin	Non-synonymous	805-806	269-269	R->K
47237	47237	A	G	Substitution			E1	E2	PA0041		probable hemagglutinin	Non-synonymous	4324-4324	1442-1442	T->A
47562	47562	G	C	Substitution			E1	E2	PA0041		probable hemagglutinin	Non-synonymous	4649-4649	1550-1550	G->A
110146	110147	CG	C	Deletion			E1	E2	PA0090	*clpV1*	ClpV1 family T6SS ATPase	Frameshift	1927-1927		
113827	113827	C	T	Substitution	D1	D2			PA0093	*tse6*	rhs-related protein	Non-synonymous	769-769	257-257	A->T
119887	119887	G	A	Substitution			E1	E2	PA0098		3-oxoacyl-ACP synthase	Non-synonymous	761-761	254-254	R->Q
148955	148955	C	T	Substitution		D2			PA0130	*bauC*	3-Oxopropanoate dehydrogenase	Non-synonymous	100-100	34-34	V->I
**163308**	**163308**	**C**	**T**	**Substitution**	**D1**	**D2**	**E1**	**E2**				**Intergenic**	**promoter mutation 55-bp upstream of PA0143**		
261683	261683	G	A	Substitution		D2			PA0231	*pcaD*	beta-ketoadipate enol-lactone hydrolase	Non-synonymous	505-505	169-169	V->I
266010	266010	C	T	Substitution	D1	D2			PA0235	*pcaK*	4-hydroxybenzoate transporter PcaK	Non-synonymous	301-301	101-101	V->I
278751	278751	C	T	Substitution	D1	D2			PA0246		putative transporter-like membrane protein	Non-synonymous	649-649	217-217	A->T
287579	287579	A	G	Substitution	D1	D2			PA0256		hypothetical protein	Non-synonymous	542-542	181-181	I->T
308726	308726	C	T	Substitution	D1	D2			PA0273		putative transporter-like membrane protein	Non-synonymous	367-367	123-123	A->T
318072	318072	C	T	Substitution		D2			PA0284		hypothetical protein	Non-synonymous	77-77	26-26	G->D
326727	326727	A	G	Substitution			E1	E2				Intergenic	located in 611-bp region between PA0290 and PA0291		
330604	330604	A	G	Substitution		D2			PA0293	*aguB*	N-carbamoylputrescine amidohydrolase	Non-synonymous	268-268	90-90	F->L
381206	381206	A	G	Substitution	D1	D2			PA0338		cyclic di-GMP signal transduction protein	Non-synonymous	304-304	102-102	I->V
396803	396803	T	C	Substitution	D1	D2			PA0353	*ilvD*	dihydroxy-acid dehydratase	Non-synonymous	1093-1093	365-365	T->A
406026	406026	C	T	Substitution			E1	E2	PA0361		putative gamma-glutamyltranspeptidase	Non-synonymous	94-94	32-32	D->N
425613	425613	T	TG	Insertion			E1	E2	PA0383		hypothetical protein	Frameshift	110-111		
427273	427273	G	A	Substitution			E1	E2	PA0385		hypothetical protein	Non-synonymous	233-233	78-78	A->V
523732	523732	T	C	Substitution	D1				PA0463	*creB*	two-component response regulator CreB	Non-synonymous	479-479	160-160	L->P
544510	544510	C	CA	Insertion	D1	D2						Intergenic	located 144-bp upstream of PA0483		
545860	545860	A	G	Substitution	D1	D2						Intergenic	located in 688-bp region between PA0484 and PA0485		
580776	580776	A	G	Substitution				E2	PA0520	*nirQ*	regulatory protein NirQ	Non-synonymous	461-461	154-154	K->R
598032	598032	C	T	Substitution			E1	E2	PA0539		EamA domain-containing protein[f]	Stop gained	325-325	109-109	Q->Stop
604691	604691	A	G	Substitution	D1	D2			PA0546	*metK*	methionine adenosyltransferase	Non-synonymous	206-206	69-69	L->P
631601	631602	GC	G	Deletion			E1	E2	PA0575	*rmcA*	redox regulator of c-di-GMP RmcA	Frameshift	2663-2663		
678601	678601	A	G	Substitution		D2			PA0619		probable bacteriophage protein	Non-synonymous	316-316	106-106	T->A
682301	682301	A	G	Substitution			E1	E2	PA0622		probable bacteriophage protein	Non-synonymous	908-908	303-303	D->G
698010	698010	A	G	Substitution	D1	D2			PA0641		probable bacteriophage protein	Non-synonymous	2929-2929	977-977	T->A
699304	699304	C	T	Substitution	D1	D2			PA0642		hypothetical protein	Non-synonymous	372-372	125-125	P->L
716607	716608	AG	A	Deletion			E1		PA0663		hypothetical protein	Frameshift	598-598		
721718	721718	A	G	Substitution	D1	D2						Intergenic	located in 538-bp region between PA0668 and PA0668.1		
721725	721725	C	T	Substitution	D1	D2						Intergenic	located in 538-bp region between PA0668 and PA0668.1		
721740	721740	C	T	Substitution	D1	D2						Intergenic	located in 538-bp region between PA0668 and PA0668.1		
732174	732174	T	TG	Insertion			E1		PA0671		hypothetical protein	Frameshift	479-480		
740432	740432	A	G	Substitution			E1	E2	PA0683	*hxcY*	type II secretion system protein	Non-synonymous	230-230	77-77	H->R
754530	754531	AG	A	Deletion	D1	D2			PA0690	*pdtA*	phosphate depletion regulated TPS partner A PdtA	Frameshift	4575-4575		
783532	783534	ACC	A	Deletion		D2						Intergenic	located 19-bp upstream of PA0711		
788414	788416	TGG	T	Deletion			E1	E2				Intergenic	located in 290-bp region between PA0716 and PA0716.1		
818399	818399	A	G	Substitution			E1	E2	PA0750	*ung*	uracil-DNA glycosylase	Non-synonymous	397-397	133-133	T->A
819327	819327	A	G	Substitution			E1	E2	PA0751		putative membrane-associated ammonia monooxygenase	Non-synonymous	536-536	179-179	V->A

821775	821775	A	G	Substitution			E1		PA0753		putative tricarboxylate transport protein TctB	Non-synonymous	79-79	27-27	W->R
831466	831467	AT	A	Deletion			E1	E2	PA0762	*algU*	sigma factor AlgU	Frameshift	167-167		
831800	831800	C	T	Substitution			E1		PA0762	*algU*	sigma factor AlgU	Non-synonymous	500-500	167-167	P->L
907573	907573	A	G	Substitution	D1							Intergenic	located 21-bp upstream of PA0833		
914839	914839	A	G	Substitution			E1	E2	PA0839		putative TetR family transcriptional regulator	Non-synonymous	428-428	143-143	Q->R
916121	916121	C	T	Substitution	D1				PA0840		probable oxidoreductase	Non-synonymous	1079-1079	360-360	P->L
920858	920858	T	C	Substitution	D1	D2			PA0844	*plcH*	hemolytic phospholipase C precursor	Non-synonymous	593-593	198-198	D->G
921153	921153	A	G	Substitution	D1	D2			PA0844	*plcH*	hemolytic phospholipase C precursor	Non-synonymous	298-298	100-100	F->L
938299	938299	C	CG	Insertion			E1		PA0860		probable ATP-binding/permease fusion ABC transporter	Frameshift	268-269		
946252	946252	A	G	Substitution			E1	E2	PA0865	*hpd*	4-hydroxyphenylpyruvate dioxygenase	Non-synonymous	419-419	140-140	D->G
950377	950377	A	G	Substitution	D1	D2			PA0869	*pbpG*	D-alanyl-D-alanine-endopeptidase	Non-synonymous	272-272	91-91	I->T
974696	974696	C	T	Substitution				E2	PA0891	*aotO*	succinylglutamate desuccinylase/aspartoacylase	Non-synonymous	215-215	72-72	P->L
994619	994620	CG	C	Deletion			E1	E2	PA0910	*alpD*	hypothetical protein	Frameshift	478-478		
**1014333**	**1014333**	**C**	**T**	**Substitution**	D1	D2	E1	E2	**PA0928**	***gacS***	**sensor/response regulator hybrid**	**Non-synonymous**	**1420-1420**	**474-474**	**G->S**
1016325	1016326	CG	C	Deletion	D1	D2			PA0929	*pirR*	two-component response regulator	Frameshift	389-389		
1085710	1085710	C	T	Substitution	D1	D2			PA1002	*phnB*	anthranilate synthase component II	Non-synonymous	169-169	57-57	P->S
1086989	1086989	C	T	Substitution			E1	E2	PA1003	*mvfR/pqsR*	transcriptional regulator MvfR	Non-synonymous	107-107	36-36	S->N
1092520	1092521	TG	T	Deletion			E1	E2	PA1009		putative glycine cleavage system transcriptional repressor	Frameshift	547-547		
1114264	1114264	A	AG	Insertion			E1		PA1027	*amaB*	delta1-Piperideine-6-carboxylate dehydrogenase	Frameshift	1136-1137		
1114894	1114895	CT	C	Deletion	D1				PA1028	*amaA*	L-Pipecolate oxidase	Frameshift	122-122		
1124912	1124912	C	T	Substitution	D1				PA1037	*yicG*	membrane protein[f]	Non-synonymous	554-554	185-185	R->H
1188251	1188251	C	T	Substitution			E1	E2	PA1097	*fleQ*	transcriptional regulator FleQ	Non-synonymous	665-665	222-222	A->V
1194861	1194861	C	T	Substitution	D1	D2			PA1102	*fliG*	flagellar motor switch protein FliG	Stop gained	655-655	219-219	Q->Stop
1228274	1228274	C	T	Substitution	D1	D2			PA1137		probable oxidoreductase	Non-synonymous	925-925	309-309	A->T
1275159	1275159	C	T	Substitution	D1	D2			PA1174	*napA*	periplasmic nitrate reductase protein NapA	Non-synonymous	640-640	214-214	A->T
1296902	1296902	T	C	Substitution			E1	E2	PA1195	*ddaH*	dimethylarginine dimethylaminohydrolase DdaH	Non-synonymous	197-197	66-66	D->G
1324593	1324593	C	G	Substitution				E2				Intergenic	located in 682-bp region between PA1221 and PA1222		
1329453	1329453	G	A	Substitution	D1	D2			PA1227		hypothetical protein	Non-synonymous	262-262	88-88	E->K
1330619	1330619	C	CG	Insertion			E1	E2	PA1229	*yeaM*	probable transcriptional regulator	Frameshift	764-765		
1349579	1349579	T	C	Substitution	D1	D2			PA1245	*aprX*	alkaline protease secretion protein	Non-synonymous	164-164	55-55	V->A
1362173	1362173	C	T	Substitution	D1				PA1253	*lhpG*	alpha-ketoglutaric semialdehyde dehydrogenase, LhpG	Non-synonymous	106-106	36-36	A->T
1364561	1364562	CG	C	Deletion			E1	E2	PA1256	*lhpO*	ABC transporter ATP-binding protein, LhpO	Frameshift	660-660		
1386876	1386876	A	G	Substitution			E1	E2	PA1275	*cobD*	cobalamin biosynthetic protein CobD	Non-synonymous	554-554	185-185	Y->C
1442310	1442310	A	G	Substitution		D2			PA1330		probable short-chain dehydrogenase	Non-synonymous	242-242	81-81	N->S
1452387	1452387	A	G	Substitution	D1	D2			PA1338	*ggt*	gamma-glutamyltranspeptidase precursor	Non-synonymous	1006-1006	336-336	Y->H
1462180	1462181	AG	A	Deletion	D1				PA1347		probable transcriptional regulator	Frameshift	450-450		
1467178	1467178	A	AC	Insertion			E1	E2	PA1352		Major facilitator family transporter	Frameshift	142-143		
1527669	1527669	A	G	Substitution	D1	D2			PA1405		probable helicase	Non-synonymous	479-479	160-160	E->G
1545019	1545019	C	T	Substitution			E1	E2	PA1419		cytosine/purines uracil thiamine allantoin permease	Non-synonymous	800-800	267-267	G->D
1553813	1553813	C	T	Substitution	D1				PA1428a			Non-synonymous	343-343	115-115	G->S

1567344	1567344	C	T	Substitution			E1	E2	PA1437		putative two-component response regulator	Non-synonymous	164-164	55-55	P->L
1578709	1578709	C	T	Substitution			E1	E2	PA1450		TPM-phosphatase domain-containing protein[f]	Non-synonymous	1163-1163	388-388	S->F
1597651	1597651	T	C	Substitution	D1	D2			PA1469		hypothetical protein	Non-synonymous	287-287	96-96	V->A
1622311	1622311	C	T	Substitution			E1	E2	PA1494	*muiA*	mucoidy inhibitor gene A	Non-synonymous	817-817	273-273	A->T
1634038	1634038	A	G	Substitution			E1	E2	PA1504		probable transcriptional regulator	Non-synonymous	455-455	152-152	L->P
1651594	1651594	T	C	Substitution			E1	E2	PA1519		xanthine/uracil permeases family protein	Non-synonymous	305-305	102-102	N->S
1651732	1651732	T	A	Substitution	D1	D2			PA1519		xanthine/uracil permeases family protein	Non-synonymous	167-167	56-56	N->I
1661745	1661745	C	T	Substitution	D1				PA1527		putative chromosome partition protein	Stop gained	334-334	112-112	Q->Stop
1665861	1665861	C	T	Substitution	D1	D2			PA1528	*zipA*	cell division protein ZipA	Non-synonymous	797-797	266-266	T->I
1680424	1680424	G	GC	Insertion			E1	E2				Intergenic	located 227-bp upstream of PA1542		
1699502	1699502	A	G	Substitution			E1	E2	PA1561	*aer*	aerotaxis receptor Aer	Non-synonymous	446-446	149-149	V->A
1721923	1721923	G	A	Substitution			E1	E2	PA1583	*sdhA*	succinate dehydrogenase (A subunit)	Non-synonymous	428-428	143-143	R->H
1738203	1738203	A	G	Substitution			E1	E2	PA1596	*htpG*	heat shock protein HtpG	Non-synonymous	668-668	223-223	D->G
1745954	1745954	A	AGG	Insertion	D1	D2			PA1602		probable oxidoreductase	Frameshift	70-71		
1762246	1762246	C	T	Substitution			E1	E2	PA1617		probable AMP-binding enzyme	Stop gained	1534-1534	512-512	Q->Stop
1764517	1764517	C	T	Substitution			E1					Intergenic	located 19-bp upstream of PA1621		
1777890	1777890	T	C	Substitution			E1	E2	PA1634	*kdpB*	potassium-transporting ATPase, B chain	Non-synonymous	140-140	47-47	L->P
1793371	1793371	A	AG	Insertion				E2	PA1647		probable sulfate transporter	Frameshift	1369-1370		
1795533	1795533	C	T	Substitution			E1	E2	PA1648		probable oxidoreductase	Non-synonymous	355-355	119-119	A->T
1839673	1839673	C	T	Substitution			E1	E2	PA1689		putative sulfatase	Non-synonymous	1414-1414	472-472	P->S
1844992	1844992	T	TG	Insertion	D1	D2			PA1695	*pscP*	translocation protein in type III secretion	Frameshift	261-262		
1877311	1877311	G	GC	Insertion	D1	D2			PA1734		cytochrome c family protein	Frameshift	98-99		
1896891	1896891	A	G	Substitution			E1		PA1757	*thrH*	homoserine kinase	Non-synonymous	262-262	88-88	I->V
1928157	1928157	C	T	Substitution			E1	E2	PA1781	*nirB*	assimilatory nitrite reductase large subunit	Non-synonymous	470-470	157-157	G->D
1932203	1932203	C	T	Substitution			E1	E2	PA1784		putative alginate lyase	Non-synonymous	622-622	208-208	V->M
1975431	1975431	C	T	Substitution			E1		PA1818	*cadA*	lysine decarboxylase	Non-synonymous	611-611	204-204	P->L
1983636	1983636	A	G	Substitution	D1	D2			PA1824		probable membrane transporter protein[f]	Non-synonymous	653-653	218-218	V->A
2043443	2043443	G	A	Substitution	D1	D2			PA1874		probable surface adhesion protein	Non-synonymous	7003-7003	2335-2335	G->S
2045119	2045119	A	G	Substitution				E2	PA1875	*opmL*	probable outer membrane protein precursor	Non-synonymous	1273-1273	425-425	S->G
2052577	2052577	C	T	Substitution			E1	E2	PA1881		probable oxidoreductase	Non-synonymous	34-34	12-12	V->I
2083822	2083822	G	A	Substitution	D1	D2			PA1910	*femA*	ferric-mycobactin receptor, FemA	Non-synonymous	446-446	149-149	A->V
2097333	2097333	T	TG	Insertion	D1	D2						Intergenic	located in 168-bp region between PA1921 and PA1922		
2118685	2118685	T	TG/TGG	Insertion			E1	E2	PA1937		conserved hypothetical protein	Frameshift	101-102		
**2134424**	**2134424**	**C**	**T**	**Substitution**	**D1**	**D2**	**E1**	**E2**	**PA1949**	***rbsR***	**ribose operon repressor RbsR**	**Non-synonymous**	**32-32**	**11-11**	**A->V**
2147824	2147824	C	T	Substitution			E1	E2	PA1964	*ybiT*	probable ATP-binding component of ABC transporter	Non-synonymous	703-703	235-235	A->T
2156286	2156286	C	T	Substitution		D2			PA1973	*pqqF*	pyrroloquinoline quinone biosynthesis protein F	Non-synonymous	913-913	305-305	R->W
2175755	2175755	C	T	Substitution			E1	E2	PA1990	*pqqH*	dipeptidyl aminopeptidase/acylaminoacyl peptidase	Non-synonymous	694-694	232-232	R->C
2177604	2177604	G	A	Substitution			E1	E2	PA1991		probable iron-containing alcohol dehydrogenase	Non-synonymous	631-631	211-211	G->S
2190874	2190874	G	GA	Insertion	D1							Intergenic	located 20-bp upstream of PA2003		
2206813	2206813	G	A	Substitution	D1							Intergenic	located 7-bp upstream of PA2016		
2211750	2211750	A	G	Substitution	D1	D2			PA2019	*mexX*	Resistance-Nodulation-Cell Division (RND) multidrug efflux membrane fusion protein MexX precursor	Non-synonymous	763-763	255-255	Y->H
2217007	2217007	T	C	Substitution	D1	D2			PA2025	*gor*	glutathione reductase	Non-synonymous	320-320	107-107	V->A
2221724	2221724	C	T	Substitution			E1	E2	PA2032	*yjiR*	putative GntR family transcriptional regulator	Non-synonymous	388-388	130-130	R->W

2237474	2237474	A	G	Substitution			E1	E2	PA2044		putative cysteine proteases	Non-synonymous	893-893	298-298	V->A
2264100	2264100	A	G	Substitution			E1	E2	PA2065	*pcoA*	copper resistance protein A precursor	Non-synonymous	878-878	293-293	V->A
2286271	2286271	T	C	Substitution	D1	D2			PA2078		(7S,10S)-hydroperoxide diol synthase	Non-synonymous	967-967	323-323	T->A
2318840	2318840	T	TG	Insertion	D1	D2			PA2108	*PA2108*	probable decarboxylase	Frameshift	46-47		
2322453	2322453	A	AG	Insertion	D1				PA2111		allophanate hydrolase subunit 1	Frameshift	328-329		
2360207	2360207	A	G	Substitution	D1				PA2144	*glgP*	glycogen phosphorylase	Non-synonymous	1844-1844	615-615	N->S
2361536	2361536	A	G	Substitution	D1	D2						Intergenic	located 170-bp upstream of PA2146		
2411751	2411751	A	AC	Insertion	D1	D2			PA2192		hypothetical protein	Frameshift	43-44		
2437723	2437723	G	T	Substitution			E1	E2	PA2217		probable aldehyde dehydrogenase	Non-synonymous	296-296	99-99	R->L
2471427	2471427	G	A	Substitution				E2	PA2244	*pslN*	DNA topoisomerase	Non-synonymous	353-353	118-118	R->Q
2494040	2494040	C	T	Substitution	D1	D2			PA2265	*gad*	gluconate dehydrogenase	Non-synonymous	104-104	35-35	A->V
2515309	2515309	G	GC	Insertion			E1	E2	PA2286		putative peptidase, M48 domain-containing protein[f]	Frameshift	1468-1469		
2568824	2568824	C	G	Substitution	D1							Intergenic	located in 643-bp region between PA2326 and PA2327		
2568911	2568911	G	A	Substitution	D1							Intergenic	located in 643-bp region between PA2326 and PA2327		
2574930	2574930	C	T	Substitution			E1	E2	PA2333		probable sulfatase	Non-synonymous	1063-1063	355-355	G->S
2582731	2582731	A	G	Substitution				E2	PA2338	*mtlE*	probable binding protein component of ABC maltose/mannitol transporter	Non-synonymous	635-635	212-212	Q->R
2582952	2582952	G	GC	Insertion	D1	D2			PA2338	*mtlE*	probable binding protein component of ABC maltose/mannitol transporter	Frameshift	856-857		
2586319	2586319	G	GC	Insertion	D1				PA2341	*mtlK*	probable ATP-binding component of ABC maltose/mannitol transporter	Frameshift	1021-1022		
2629038	2629038	G	A	Substitution	D1	D2			PA2377		hypothetical protein	Non-synonymous	814-814	272-272	G->S
2648395	2648395	G	A	Substitution	D1				PA2392	*pvdP*	pyoverdine synthesis protein	Non-synonymous	16-16	6-6	R->C
2708835	2708835	T	C	Substitution	D1	D2	E1	E2	PA2424	*pvdL*	non-ribosomal peptide synthetase PvdL	Non-synonymous	11860-11860	3954-3954	I->V
**2808355**	**2808355**	**T**	**C**	**Substitution**			**E1**	**E2**	**PA2492**	***mexT***	**transcriptional regulator MexT**	**Non-synonymous**	**887-887**	**296-296**	**L->P**
**2812077**	**2812077**	**G**	**A**	**Substitution**	**D1**	**D2**	**E1**	**E2**	**PA2494**	***mexF***	**Resistance-Nodulation-Cell Division (RND) multidrug efflux transporter MexF**	**Non-synonymous**	**2069-2069**	**690-690**	**R->H**
2867736	2867736	G	A	Substitution		D2			PA2540		lysophospholipase	Non-synonymous	1567-1567	523-523	P->S
2875586	2875586	G	A	Substitution		D2						Intergenic	located 23-bp upstream of PA2543		
2902224	2902224	A	G	Substitution	D1	D2			PA2567		putative GAF sensor-containing diguanylate cyclase/phosphodiesterase	Non-synonymous	7-7	3-3	T->A
2905159	2905160	CT	C	Deletion	D1	D2						Intergenic	located in the region bewteen PA2570 and PA2570.1		
2939341	2939341	G	A	Substitution			E1	E2	PA2596		putative periplasmic aliphatic sulfonate-binding protein	Non-synonymous	199-199	67-67	L->F
2945601	2945601	C	CGG	Insertion		D2	E1	E2	PA2602		3-mercaptopropionate dioxygenase	Frameshift	338-339		
2963796	2963796	T	C	Substitution			E1	E2	PA2620	*clpA*	ATP-binding protease component ClpA	Non-synonymous	784-784	262-262	T->A
2992044	2992044	G	A	Substitution	D1	D2			PA2645	*nuoJ*	NADH dehydrogenase I chain J	Non-synonymous	43-43	15-15	A->T
2998057	2998057	G	A	Substitution	D1				PA2650	*ybaJ*	putative methyltransferase	Non-synonymous	218-218	73-73	G->D
3032471	3032471	G	A	Substitution			E1		PA2684	*tse5*	rhs-like protein	Stop gained	3232-3232	1078-1078	Q->Stop
3040399	3040400	CG	C	Deletion			E1	E2	PA2688	*pfeA*	Ferric enterobactin receptor, outer membrane protein PfeA precursor	Frameshift	159-159		
3114142	3114142	G	A	Substitution	D1	D2						Intergenic	located in 461-bp region between PA2751 and PA2752		
3118193	3118193	C	T	Substitution	D1				PA2757		transmembrane pair domain-containing protein	Start lost	1-1	1-1	V->M
3119635	3119635	G	A	Substitution			E1		PA2759		putative lipoprotein	Non-synonymous	74-74	25-25	P->L
3127469	3127469	G	A	Substitution		D2			PA2768		hypothetical protein	Non-synonymous	244-244	82-82	A->T
3135963	3135964	CG	C	Deletion	D1	D2			PA2777	*yfdC*	formate/nitrate transporter	Frameshift	37-37		
3160161	3160162	AC	A	Deletion			E1	E2	PA2804		putative phosphohydrolase	Frameshift	497-497		
3161351	3161351	A	G	Substitution			E1	E2	PA2806	*yqcD*	7-cyano-7-deazaguanine reductase	Non-synonymous	131-131	44-44	L->P

3194752	3194752	C	T	Substitution		D2			PA2840	*deaD*	probable ATP-dependent RNA helicase	Non-synonymous	838-838	280-280	G->S
3267259	3267259	G	A	Substitution	D1				PA2911		probable TonB-dependent receptor	Non-synonymous	1412-1412	471-471	R->H
3274376	3274376	T	C	Substitution				E2	PA2919		hypothetical protein	Non-synonymous	49-49	17-17	F->L
3282931	3282931	A	G	Substitution			E1	E2	PA2927		hypothetical protein	Non-synonymous	944-944	315-315	E->G
3283331	3283331	G	GCC	Insertion			E1	E2				Intergenic	located in 54-bp region between PA2927 and PA2928		
3288876	3288876	G	A	Substitution	D1				PA2933		probable major facilitator superfamily (MFS) transporter	Non-synonymous	362-362	121-121	G->D
3354563	3354563	G	A	Substitution			E1	E2	PA2997	*nqrC*	Na+-translocating NADH:ubiquinone oxidoreductase subunit Nrq3	Non-synonymous	392-392	131-131	T->I
3381043	3381043	T	C	Substitution	D1	D2			PA3019	*uup*	probable ATP-binding component of ABC transporter	Non-synonymous	695-695	232-232	N->S
3394105	3394105	G	GCC	Insertion	D1	D2			PA3030	*mobA*	molybdopterin-guanine dinucleotide biosynthesis protein MobA	Frameshift	18-19		
3398922	3398922	G	GC	Insertion			E1		PA3036		putative oxidoreductase	Frameshift	206-207		
3417078	3417078	T	C	Substitution			E1	E2				Intergenic	located 35-bp upstream of PA3053		
3432467	3432468	GC	G	Deletion	D1	D2			PA3064	*pelA*	biofilm formation protein PelA	Frameshift	1424-1424		
3445522	3445522	T	C	Substitution	D1	D2			PA3073		hypothetical protein	Non-synonymous	287-287	96-96	V->A
3451617	3451617	G	A	Substitution	D1				PA3078	*cprS*	sensor histidine kinase	Non-synonymous	112-112	38-38	A->T
3467219	3467219	G	A	Substitution				E2	PA3089		hypothetical protein	Stop gained	135-135	45-45	W->Stop
3480355	3480355	T	C	Substitution	D1	D2			PA3101	*xcpT*	general secretion pathway protein G	Non-synonymous	338-338	113-113	Q->R
3493764	3493764	G	A	Substitution	D1	D2						Intergenic	located 191-bp upstream of PA3112		
3498918	3498918	G	A	Substitution	D1				PA3116		probable aspartate-semialdehyde dehydrogenase	Non-synonymous	467-467	156-156	A->V
3512594	3512594	G	A	Substitution	D1	D2			PA3131	*edaB*	probable aldolase	Non-synonymous	448-448	150-150	P->S
3514707	3514707	C	T	Substitution	D1	D2						Intergenic	located 142-bp upstream of PA3133		
3541386	3541386	T	C	Substitution				E2	PA3157	*wbpC*	probable acetyltransferase	Non-synonymous	1286-1286	429-429	Y->C
3541554	3541554	G	G/GC	Insertion			E1	E2	PA3157	*wbpC*	probable acetyltransferase	Frameshift	1117-1118		
3556143	3556143	G	A	Substitution	D1	D2			PA3167	*serC*	3-phosphoserine aminotransferase	Non-synonymous	197-197	66-66	S->L
3570169	3570169	A	G	Substitution				E2	PA3179	*yciL*	ribosomal large subunit pseudouridine synthase B	Non-synonymous	98-98	33-33	V->A
3597948	3597948	G	GC	Insertion		D2			PA3208	*ydjA*	putative NAD(P)H nitroreductase YdjA	Frameshift	391-392		
3619006	3619006	T	C	Substitution	D1	D2			PA3232		probable nuclease	Non-synonymous	614-614	205-205	K->R
3631823	3631823	C	T	Substitution			E1	E2	PA3244	*minD*	cell division inhibitor MinD	Non-synonymous	206-206	69-69	T->I
3645915	3645915	G	A	Substitution	D1	D2			PA3258		diguanylate phosphodiesterase	Non-synonymous	817-817	273-273	A->T
3663717	3663717	G	A	Substitution			E1		PA3272		probable ATP-dependent DNA helicase	Non-synonymous	4139-4139	1380-1380	A->V
3700015	3700015	T	C	Substitution	D1				PA3301		putative lysophospholipase	Non-synonymous	647-647	216-216	V->A
3723159	3723159	G	A	Substitution	D1	D2			PA3320		hypothetical protein	Non-synonymous	287-287	96-96	A->V
3747910	3747910	T	TG	Insertion		D2			PA3339	*plpD*	patatin-like protein, PlpD	Frameshift	386-387		
3801642	3801642	G	A	Substitution		D2			PA3395	*nosY*	nitrous oxide reductase maturation protein NosY	Non-synonymous	539-539	180-180	G->D
3809400	3809400	G	A	Substitution			E1	E2	PA3404	*opmM*	probable outer membrane protein precursor	Non-synonymous	1214-1214	405-405	A->V
3816144	3816144	G	A	Substitution	D1				PA3408	*hasR*	heme uptake outer membrane receptor HasR precursor	Non-synonymous	1193-1193	398-398	A->V
3884014	3884014	G	A	Substitution			E1	E2	PA3471	*sfcA*	probable malic enzyme	Non-synonymous	1241-1241	414-414	P->L
3889582	3889582	T	C	Substitution		D2			PA3476	*rhlI*	autoinducer synthesis protein RhlI	Non-synonymous	163-163	55-55	T->A
**3906126**	**3906128**	**AGG**	**A/AG**	**Deletion**	**D1**	**D2**			**PA3488**	***tli5***	**T6SS-associated effector immunity protein**	**Frameshift**	73-74		
**3906334**	**3906334**	**C**	**CA**	**Insertion**			**E1**	**E2**	**PA3488**	***tli5***	**T6SS-associated effector immunity protein**	**Frameshift**	280-281		
3908772	3908772	G	A	Substitution	D1				PA3491	*rnfC*	probable ferredoxin	Non-synonymous	361-361	121-121	A->T
3934712	3934712	G	GC	Insertion		D2			PA3517		probable lyase	Frameshift	38-39		
3947041	3947041	G	GC	Insertion			E1	E2				Intergenic	located 54-bp upstream of PA3527		
3947621	3947621	T	C	Substitution		D2			PA3527	*pyrC*	dihydroorotase	Non-synonymous	527-527	176-176	I->T
**3957397**	**3957397**	**C**	**T**	**Substitution**	**D1**	**D2**	**E1**	**E2**	**PA3535**	***eprS***	**probable serine protease**	**Stop gained**	2491-2491	831-831	Q->Stop

3971160	3971160	T	C	Substitution	D1	D2			PA3545	*algG*	alginate-c5-mannuronan-epimerase AlgG	Non-synonymous	1219-1219	407-407	W->R
**3974147**	**3974147**	**G**	**A**	**Substitution**	**D1**	**D2**	**E1**	**E2**				**Intergenic**	**located 212-bp upstream of PA3548**		
3976828	3976828	A	G	Substitution			E1	E2	PA3549	*algJ*	alginate o-acetyltransferase AlgJ	Non-synonymous	893-893	298-298	Y->C
3981001	3981001	G	A	Substitution	D1				PA3552	*arnB*	UDP-4-amino-4-deoxy-L-arabinose--oxoglutarate aminotransferase	Non-synonymous	1142-1142	381-381	R->H
3981135	3981135	T	C	Substitution	D1				PA3553	*arnC*	glycosyl transferase ArnC	Non-synonymous	131-131	44-44	L->P
3998327	3998327	G	A	Substitution	D1	D2			PA3567		probable oxidoreductase	Non-synonymous	191-191	64-64	G->D
4000391	4000391	T	C	Substitution	D1				PA3568	*ymmS*	probable acetyl-coa synthetase	Non-synonymous	704-704	235-235	Y->C
4002895	4002895	T	C	Substitution	D1	D2			PA3570	*mmsA*	methylmalonate-semialdehyde dehydrogenase	Non-synonymous	707-707	236-236	H->R
4014849	4014849	G	A	Substitution	D1	D2			PA3582	*glpK*	glycerol kinase	Non-synonymous	1165-1165	389-389	A->T
4015416	4015416	G	GC	Insertion	D1				PA3583	*glpR*	glycerol-3-phosphate regulon repressor	Frameshift	10-10		
**4056590**	**4056592**	**TGC**	**T**	**Deletion**	**D1**	**D2**	**E1**	**E2**	**PA3620**	***mutS***	**DNA mismatch repair protein MutS**	**Frameshift**	**2067-2068**		
4061772	4061772	T	C	Substitution	D1				PA3626	*ygbO*	tRNA pseudouridine synthase D	Non-synonymous	658-658	220-220	S->G
4062198	4062198	T	C	Substitution			E1	E2	PA3626	*ygbO*	tRNA pseudouridine synthase D	Non-synonymous	232-232	78-78	S->G
4063955	4063955	T	C	Substitution		D2			PA3629	*adhC*	alcohol dehydrogenase class III	Non-synonymous	1033-1033	345-345	T->A
4064188	4064188	T	C	Substitution			E1		PA3629	*adhC*	alcohol dehydrogenase class III	Non-synonymous	800-800	267-267	N->S
4080297	4080297	A	G	Substitution			E1	E2				Intergenic	located 74-bp upstream of PA3641		
4087151	4087151	T	C	Substitution				E2	PA3648	*opr86*	outer membrane protein Opr86	Non-synonymous	305-305	102-102	K->R
4103569	4103569	G	A	Substitution	D1	D2						Intergenic	located 140-bp upstream of PA3663		
4129634	4129634	G	GC	Insertion				E2	PA3687	*ppc*	phosphoenolpyruvate carboxylase	Frameshift	758-759		
4179665	4179665	C	T	Substitution	D1				PA3729		conserved hypothetical protein	Non-synonymous	1678-1678	560-560	E->K
4185732	4185732	T	TC	Insertion		D2			PA3734		putative hydrolase[f]	Frameshift	211-212		
4198659	4198659	T	C	Substitution			E1	E2				Intergenic	located in 302-bp region between PA3746 and PA3747		
4204066	4204066	C	T	Substitution		D2			PA3751	*purT*	phosphoribosylglycinamide formyltransferase 2	Non-synonymous	440-440	147-147	R->H
4219532	4219532	G	A	Substitution			E1					Intergenic	located 92-bp upstream of PA3763		
4231182	4231183	GC	G	Deletion			E1		PA3773		major facilitator family transporter	Frameshift	113-113		
4238746	4238747	GC	G	Deletion		D2						Intergenic	located 61-bp upstream of PA3780		
4283969	4283969	A	G	Substitution			E1	E2	PA3826		hypothetical protein	Non-synonymous	182-182	61-61	D->G
4285091	4285091	G	A	Substitution				E2	PA3827	*lptG*	Lipopolysaccharide export system permease protein LptG	Non-synonymous	394-394	132-132	R->C
4294956	4294956	T	C	Substitution			E1					Intergenic	located in 2643-bp region between PA3835 and PA3836 (+)		
4295049	4295050	TC	T	Deletion	D1	D2						Intergenic	located in 2643-bp region between PA3835 and PA3836 (+)		
4315086	4315086	A	G	Substitution				E2	PA3855		hypothetical protein	Non-synonymous	404-404	135-135	V->A
4335151	4335151	G	A	Substitution	D1	D2			PA3871	*nifM*	probable peptidyl-prolyl cis-trans isomerase, PpiC-type	Non-synonymous	71-71	24-24	A->V
4338701	4338701	T	C	Substitution	D1				PA3875	*narG*	respiratory nitrate reductase alpha chain	Non-synonymous	3346-3346	1116-1116	T->A
4405531	4405531	G	A	Substitution	D1				PA3930	*cioA*	cyanide insensitive terminal oxidase	Non-synonymous	838-838	280-280	R->C
4416973	4416973	G	GC	Insertion			E1	E2				Intergenic	located 164-bp upstream of PA3938		
4424950	4424950	T	C	Substitution			E1	E2	PA3946	*rocS1*	two-component sensor RocS1	Non-synonymous	1901-1901	634-634	H->R
4432578	4432578	G	A	Substitution			E1					Intergenic	located 49-bp upstream of PA3951		
4462174	4462174	A	G	Substitution	D1	D2			PA3981	*ybeZ*	phosphate starvation-inducible protein PhoH	Non-synonymous	787-787	263-263	T->A
4478470	4478470	T	C	Substitution	D1				PA3997	*lipB*	lipoate-protein ligase B	Non-synonymous	158-158	53-53	K->R
4543684	4543684	G	A	Substitution	D1	D2			PA4065		putative ABC transporter permease protein	Non-synonymous	1027-1027	343-343	A->T
4545178	4545178	T	C	Substitution	D1				PA4067	*oprG*	outer membrane protein OprG precursor	Non-synonymous	572-572	191-191	V->A
4570972	4570972	T	C	Substitution			E1	E2	PA4088		glutamate-1-semialdehyde aminomutase	Non-synonymous	44-44	15-15	L->P
4591868	4591868	A	G	Substitution	D1	D2			PA4108		cyclic di-GMP phosphodiesterase	Non-synonymous	682-682	228-228	M->V
4596046	4596046	T	C	Substitution	D1	D2			PA4112		probable sensor/response regulator hybrid	Non-synonymous	4057-4057	1353-1353	S->G
4596615	4596615	A	G	Substitution	D1	D2			PA4112		probable sensor/response regulator hybrid	Non-synonymous	3488-3488	1163-1163	V->A

4598122	4598122	G	A	Substitution			E1	E2	PA4112		probable sensor/response regulator hybrid	Stop gained	1981-1981	661-661	Q->Stop
4616874	4616874	A	G	Substitution			E1	E2	PA4128	*hpcH*	putative 2,4-dihydroxyhept-2-ene-1,7-dioic acid aldolase	Non-synonymous	713-713	238-238	D->G
4660228	4660228	G	A	Substitution	D1	D2			PA4164		hypothetical protein	Non-synonymous	8-8	3-3	R->Q
4733852	4733852	G	A	Substitution	D1	D2			PA4225	*pchF*	pyochelin synthetase	Non-synonymous	2947-2947	983-983	R->C
4735313	4735313	C	T	Substitution	D1	D2			PA4225	*pchF*	pyochelin synthetase	Non-synonymous	1486-1486	496-496	G->S
4741005	4741005	T	C	Substitution				E2	PA4226	*pchE*	dihydroaeruginoic acid synthetase	Non-synonymous	107-107	36-36	D->G
4741096	4741096	C	CG	Insertion			E1	E2	PA4226	*pchE*	dihydroaeruginoic acid synthetase	Frameshift	15-16		
4752450	4752450	T	C	Substitution			E1	E2	PA4236	*katA*	catalase KatA	Non-synonymous	1259-1259	420-420	Y->C
4773063	4773063	G	A	Substitution	D1	D2			PA4269	*rpoC*	DNA-directed RNA polymerase beta chain	Non-synonymous	3416-3416	1139-1139	P->L
4780070	4780070	T	C	Substitution			E1	E2	PA4270	*rpoB*	DNA-directed RNA polymerase beta chain	Non-synonymous	548-548	183-183	Y->C
4815802	4815802	T	C	Substitution			E1	E2	PA4292		probable phosphate transporter	Non-synonymous	760-760	254-254	Y->H
4830052	4830052	G	A	Substitution	D1	D2			PA4269	*rcpC*	DNA-directed RNA polymerase beta chain	Non-synonymous	503-503	168-168	P->L
4834092	4834092	C	T	Substitution			E1	E2	PA4308		hypothetical protein	Non-synonymous	773-773	258-258	G->D
4873538	4873538	T	C	Substitution	D1				PA4344		putative hydrolase	Non-synonymous	953-953	318-318	E->G
4874042	4874042	G	A	Substitution			E1	E2	PA4344		probable hydrolase	Non-synonymous	449-449	150-150	A->V
4876117	4876117	A	G	Substitution	D1				PA4347		putative beta-lactamase	Non-synonymous	676-676	226-226	Y->H
4917423	4917423	C	T	Substitution	D1				PA4386	*groES*	co-chaperonin GroES	Non-synonymous	46-46	16-16	E->K
4926128	4926128	A	G	Substitution				E2	PA4396		putative two-component response regulator	Non-synonymous	230-230	77-77	D->G
4942993	4942993	G	A	Substitution			E1	E2	PA4410	*ddlB*	D-alanine--D-alanine ligase	Non-synonymous	644-644	215-215	P->L
4957778	4957778	T	C	Substitution	D1	D2			PA4423	*yraM*	putative lipoprotein	Non-synonymous	70-70	24-24	S->P
4962215	4962215	G	A	Substitution			E1	E2				Intergenic	located 28-bp upstream of PA4428		
4965568	4965568	T	C	Substitution	D1	D2			PA4433	*rplM*	50S ribosomal protein L13	Non-synonymous	377-377	126-126	K->R
4971047	4971047	G	A	Substitution	D1	D2			PA4438	*yhcM*	putative ATPase	Non-synonymous	845-845	282-282	A->V
4977785	4977785	G	GC	Insertion		D2						Intergenic	located 84-bp upstream of PA4444		
4985364	4985364	T	C	Substitution		D2			PA4450	*murA*	UDP-N-acetylglucosamine 1-carboxyvinyltransferase	Non-synonymous	107-107	36-36	D->G
4990271	4990271	G	A	Substitution		D2			PA4457	*kdsD*	arabinose-5-phosphate isomerase KdsD	Non-synonymous	967-967	323-323	A->T
4996759	4996759	G	A	Substitution			E1	E2	PA4467		zinc/iron permease	Non-synonymous	662-662	221-221	A->V
5008510	5008510	A	G	Substitution			E1	E2	PA4476		hypothetical protein	Non-synonymous	119-119	40-40	V->A
5026900	5026900	G	A	Substitution	D1	D2			PA4491	*magB*	hypothetical protein	Non-synonymous	484-484	162-162	R->C
**5036675**	**5036675**	**A**	**AC**	**Insertion**	**D1**	**D2**			**PA4499**	***psdR***	**pseudomonas dipeptide regulator PdsR**	**Frameshift**	**431-432**		
**5036759**	**5036759**	**T**	**C**	**Substitution**			**E1**	**E2**	**PA4499**	***psdR***	**pseudomonas dipeptide regulator PdsR**	**Non-synonymous**	**515-515**	**172-172**	**V->A**
5048637	5048637	G	A	Substitution	D1				PA4510		putative allophanate hydrolase subunit 1	Non-synonymous	251-251	84-84	A->V
5073748	5073748	T	C	Substitution	D1	D2			PA4529	*coaE*	dephosphocoenzyme A kinase	Non-synonymous	185-185	62-62	L->P
**5095955**	**5095955**	**T**	**C**	**Substitution**	**D1**	**D2**	**E1**	**E2**	**PA4547**	***pilR***	**two-component response regulator PilR**	**Non-synonymous**	**971-971**	**324-324**	**L->P**
5101022	5101023	GC	G	Deletion			E1	E2	PA4554	*pilY1*	type IV pilus assembly protein PilY1	Frameshift	341-341		
5101644	5101644	G	A	Substitution	D1				PA4554	*pilY1*	type IV fimbrial biogenesis protein PilY1	Stop gained	962-962	321-321	W->Stop
5115713	5115713	G	A	Substitution	D1	D2			PA4566	*obg*	GTP-binding protein Obg	Non-synonymous	178-178	60-60	R->C
5116811	5116811	T	TC	Insertion			E1					Intergenic	located 54-bp upstream of PA4569		
5146358	5146358	G	GC	Insertion				E2	PA4594		probable ATP-binding component of ABC transporter	Frameshift	45-46		
5204959	5204959	A	G	Substitution	D1	D2			PA4636		phospholipid/glycerol acyltransferase	Non-synonymous	32-32	11-11	Y->C
5210533	5210534	GC	G	Deletion	D1							Intergenic	located 84-bp upstream of PA4641		
5210533	5210533	G	GCC	Insertion		D2						Intergenic	located 83-bp upstream of PA4641		
5224501	5224501	T	C	Substitution			E1	E2				Intergenic	located 25-bp upstream of PA4656		
5248582	5248582	G	A	Substitution				E2	PA4679		hypothetical protein	Non-synonymous	191-191	64-64	P->L
**5280530**	**5280530**	**T**	**C**	**Substitution**			**E1**	**E2**	**PA4701**		**hypothetical protein**	**Non-synonymous**	**26-26**	**9-9**	**L->P**
**5280548**	**5280548**	**A**	**AC**	**Insertion**	**D1**	**D2**			**PA4701**		**hypothetical protein**	**Frameshift**	**44-45**		
5288685	5288686	AC	A	Deletion			E1	E2	PA4709	*phuS*	putative hemin degrading factor	Frameshift	351-351		

5292406	5292406	C	T	Substitution			E1	E2	PA4712		hypothetical protein	Non-synonymous	365-365	122-122	A->V
5343350	5343350	A	G	Substitution			E1	E2	PA4757	*yeaS*	amino acid transporter LysE	Non-synonymous	406-406	136-136	F->L
5360921	5360921	T	C	Substitution	D1	D2			PA4772		probable ferredoxin	Non-synonymous	2480-2480	827-827	I->T
5362903	5362903	T	C	Substitution			E1	E2	PA4774	*speE2*	putative spermidine synthase	Non-synonymous	758-758	253-253	V->A
5364492	5364492	T	C	Substitution	D1	D2			PA4776	*pmrA*	two-component regulator system response regulator PmrA	Non-synonymous	422-422	141-141	V->A
5374508	5374509	GC	G	Deletion	D1	D2			PA4786		probable short-chain dehydrogenase	Frameshift	387-387		
5383950	5383950	T	C	Substitution	D1	D2			PA4798		hypothetical protein	Non-synonymous	4-4	2-2	S->P
5388865	5388865	G	A	Substitution	D1				PA4804		Probable amino acid permease[f]	Non-synonymous	1235-1235	412-412	A->V
5411060	5411060	C	T	Substitution	D1	D2			PA4821	*dinF*	probable transporter	Non-synonymous	185-185	62-62	T->I
5411213	5411213	C	T	Substitution	D1	D2			PA4821	*dinF*	probable transporter	Non-synonymous	338-338	113-113	A->V
5412277	5412277	A	G	Substitution	D1	D2						Intergenic	located between PA4821 and PA4822		
5451957	5451957	C	T	Substitution			E1		PA4855	*purD*	phosphoribosylamine--glycine ligase	Non-synonymous	1201-1201	401-401	R->C
5452051	5452051	G	A	Substitution	D1	D2						Intergenic	located 99-bp upstream of PA4856		
5518910	5518910	T	C	Substitution			E1	E2	PA4919	*pncB1*	nicotinate phosphoribosyltransferase	Non-synonymous	428-428	143-143	V->A
5520596	5520596	G	A	Substitution			E1	E2				Intergenic	located 76-bp upstream of PA4921		
5525448	5525448	T	C	Substitution			E1	E2	PA4926		transglutaminase-like protein	Non-synonymous	458-458	153-153	D->G
5528525	5528525	C	T	Substitution			E1	E2				Intergenic	located 128-bp upstream of PA4927		
5539732	5539732	G	A	Substitution			E1	E2	PA4937	*rnr*	exoribonuclease RNase R	Non-synonymous	1679-1679	560-560	P->L
5566783	5566783	G	A	Substitution			E1	E2	PA4959	*fimX*	response regulator receiver modulated diguanylate cyclase/phosphodiesterase	Non-synonymous	1291-1291	431-431	A->T
5585563	5585563	G	GC	Insertion	D1	D2						Intergenic	located in region between PA4974 and PA4975		
5585593	5585593	G	A	Substitution	D1	D2						Intergenic	located between PA4974 and PA4975		
5617087	5617087	G	A	Substitution	D1	D2			PA4999	*waaL*	O-antigen ligase WaaL	Non-synonymous	778-778	260-260	G->R
5630039	5630039	A	G	Substitution			E1	E2	PA5011	*waaC*	heptosyltransferase I	Non-synonymous	815-815	272-272	V->A
5634350	5634350	T	C	Substitution			E1	E2	PA5014	*glnE*	glutamate-ammonia-ligase adenylyltransferase	Non-synonymous	1525-1525	509-509	S->G
5659454	5659454	G	A	Substitution			E1	E2	PA5029	*ynfL*	putative LysR family transcriptional regulator	Non-synonymous	728-728	243-243	S->L
5678066	5678066	A	G	Substitution	D1	D2			PA5041	*pilP*	type 4 fimbrial biogenesis protein PilP	Non-synonymous	371-371	124-124	V->A
5688528	5688528	G	A	Substitution	D1				PA5050	*priA*	primosome assembly protein PriA	Non-synonymous	1033-1033	345-345	A->T
5716803	5716803	A	G	Substitution	D1	D2			PA5077	*mdoH*	glucosyltransferase MdoH	Non-synonymous	53-53	18-18	L->P
5730568	5730569	GC	G	Deletion		D2			PA5090	*vgrG5*	T6SS spike protein VgrG5[f]	Frameshift	1277-1277		
5730568	5730568	G	GC	Insertion			E1		PA5090	*vgrG5*	T6SS spike protein VgrG5[f]	Frameshift	1277-1278		
5751260	5751260	G	A	Substitution			E1		PA5107	*blc*	outer membrane lipoprotein Blc	Non-synonymous	271-271	91-91	R->W
5756292	5756293	GC	G	Deletion	D1	D2						Intergenic	located 54-bp upstream of PA5112		
5757182	5757182	T	C	Substitution			E1		PA5113		hypothetical protein	Non-synonymous	560-560	187-187	D->G
5764334	5764334	T	C	Substitution			E1	E2	PA5117	*typA*	regulatory protein TypA	Non-synonymous	143-143	48-48	Q->R
5779445	5779445	G	A	Substitution				E2	PA5131	*pgm*	phosphoglycerate mutase	Non-synonymous	1312-1312	438-438	G->S
5827991	5827991	G	T	Substitution	D1	D2			PA5174	*fabY*	beta-acetoacetyl-acyl carrier protein synthase FabY	Non-synonymous	1857-1857	619-619	Q->H
5886244	5886244	T	C	Substitution	D1				PA5230	*yhhJ*	probable permease of ABC transporter	Non-synonymous	835-835	279-279	T->A
5923307	5923307	C	T	Substitution		D2			PA3385	*algZ*	alginate and motility regulator Z	Non-synonymous	1065-1065	355-355	M->I
5979929	5979929	G	A	Substitution				E2	PA5310	*ymdC*	phospholipase D/transphosphatidylase	Non-synonymous	736-736	246-246	A->T
5992564	5992564	G	A	Substitution	D1	D2			PA5322	*algC*	phosphomannomutase AlgC	Non-synonymous	1397-1397	466-466	G->D
5995936	5995936	T	TC	Insertion			E1	E2				Intergenic	located 125-bp upstream of PA5324		
5995977	5995977	G	A	Substitution			E1					Intergenic	located 59-bp upstream of PA5325		
6010455	6010455	G	A	Substitution			E1	E2	PA5342		putative transcriptional regulator	Non-synonymous	604-604	202-202	R->C
6019461	6019461	G	A	Substitution	D1							Intergenic	located 113-bp upstream of PA5351		
6064700	6064702	ACG	A	Deletion			E1	E2	PA5386	*cdhA*	carnitine dehydrogenase CdhA	Frameshift	147-148		
6073213	6073213	T	C	Substitution	D1	D2			PA5394	*cls*	cardiolipin synthase	Non-synonymous	172-172	58-58	M->V
6090085	6090085	C	T	Substitution		D2			PA5412		hypothetical protein	Non-synonymous	622-622	208-208	D->N
6095278	6095278	G	GCC	Insertion			E1	E2				Intergenic	located 85-bp upstream of PA5416		
6181642	6181642	G	A	Substitution			E1	E2				Intergenic	located 65-bp upstream of PA5489		
6191150	6191150	C	T	Substitution			E1	E2				Intergenic	located 74-bp upstream of PA5497		
6233550	6233550	G	A	Substitution			E1	E2	PA5541	*pyrQ*	dihydroorotase	Non-synonymous	433-433	145-145	V->M

6254986	6254986	C	T	Substitution	D1				PA5562	*spoOJ*	chromosome partitioning protein Spo0J	Non-synonymous	859-859	287-287	A->T

[a]Genomic location of mutation. As described in Table 1, bold text indicates loci with SNPs or in/dels shared among all four isolates from the day-160 lineages with minimum read frequencies of 90%.
[b]Nucleotide change from the PAO1 reference genome (NC_002516.2, 2020).
[c]As described at pseudomonas.com (1).
[d]SNP location relative to the start of CDS of the affected gene. If intergenic, SNP location and any annotated motifs[c] are described relative to flanking genes, with strandedness indicated where relevant.
[e]Location of SNP relative to amino acid sequence start codon.
[f]As described at uniprot.org.
