# Supplementary material for: Evolution of the Quorum Sensing Regulon in Cooperating Populations of Pseudomonas aeruginosa
Source: mBio. 2022 Feb 22;13(1):e00161-22. doi: 10.1128/mbio.00161-22 (PMC8863103; doi:10.1128/mbio.00161-22)
Supplement: FIG S1 [file mbio.00161-22-sf001.pdf]

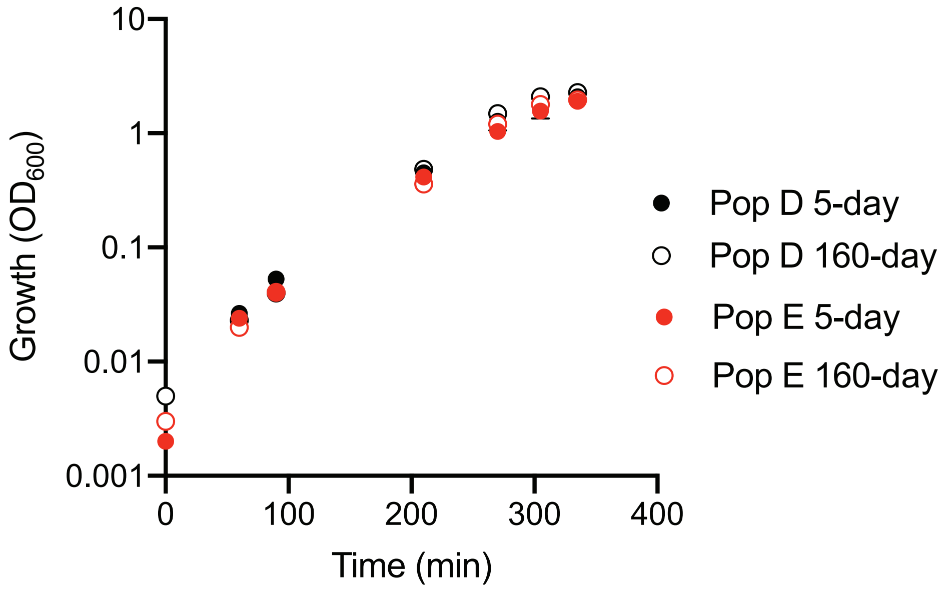

Figure S1. Growth of 5-day and 160-day populations in LB. Preparation of inocula and growth conditions were as described in the RNA-Seq Analyses section of the Materials and Methods. The data are means of two biological replicates and error bars indicate the ranges. Most error bars are smaller than the symbols.
