# Supplementary material for: Evolution of the Quorum Sensing Regulon in Cooperating Populations of Pseudomonas aeruginosa
Source: mBio. 2022 Feb 22;13(1):e00161-22. doi: 10.1128/mbio.00161-22 (PMC8863103; doi:10.1128/mbio.00161-22)
Supplement: TABLE S2 [file mbio.00161-22-st002.pdf]

Supplementary Table 2. Genes with decreased expression at day 160 in CAB-evolved Populations D and E, as compared to day 5.

	Fold Change[a]					
	Both Day 160 Lineages					
**Locus tag[b]**	**Pop D**	**Pop E**	**D only**	**E only**	**Gene name[b]**	**Description**
PA0007			-4.3			hypothetical protein
PA0012			-3.2			hypothetical protein
PA0027			-3.5			putative peptidylprolyl isomerase
PA0028			-5.5			hypothetical protein
PA0040	-4.7	-3.5				TPS family activation/secretion protein
PA0045	-3.6	-3.2				putative lipoprotein or curli production protein CsgG
PA0050	-30.6	-13.8				hypothetical protein
PA0059				-9.3	*osmC*	osmotically inducible protein OsmC
PA0062				-4.8		putative lipoprotein
PA0070	-5.3	-2.9			*tagQ1*	T6SS-associated lipoprotein TagQ
PA0072	-5.9	-8.0			*tagS1*	T6SS-associated transmembrane protein TagS
PA0073	-7.3	-7.2			*tagT1*	T6SS-associated ATP-binding protein TagT
PA0074	-4.6	-3.1			*ppkA*	serine/threonine protein kinase PpkA
PA0075			-3.9		*pppA*	serine/threonine phosphatase
PA0077			-3.8		*icmF1*	T6SS protein IcmF
PA0078			-4.7			hypothetical protein
PA0080			-3.8			hypothetical protein
PA0082			-3.1			hypothetical protein
PA0083			-6.9			hypothetical protein
PA0084			-5.2			hypothetical protein
PA0085	-13.3	-4.4			*hcp1*	protein secretion apparatus assembly protein
PA0087	-4.6	-2.9			*tssE1*	T6SS system lysozyme-like protein
PA0088	-11.0	-3.3			*tssF1*	T6SS protein TssF1
PA0089	-13.9	-4.4			*tssG1*	T6SS protein TssG1
PA0090	-5.8	-3.6			*clpV1*	T6SS protein ClpV1
PA0091	-5.5	-3.7			*vgrG1*	T6SS system protein VgrG
PA0092			-3.2			hypothetical protein
PA0093			-3.1			hypothetical protein
PA0094			-3.6			hypothetical protein
PA0097			-5.4			hypothetical protein
PA0098	-3.9	-2.9				3-oxoacyl-ACP synthase
PA0099			-3.7			hypothetical protein
PA0173			-4.5			chemotaxis response regulator protein-glutamate methylesterase
PA0174			-5.2			hypothetical protein
PA0175			-7.8			chemotaxis protein methyltransferase
PA0176			-4.8		*aer2*	aerotaxis transducer Aer2
PA0177			-5.4			purine-binding chemotaxis protein
PA0178			-4.3			two-component sensor
PA0179			-4.4			two-component response regulator
PA0188	-2.8	-5.4				methyltransferase regulatory domain-containing protein
PA0306.1	-3.9	-3.5				transcriptional regulator
PA0355				-6.9	*pfpI*	protease PfpI
PA0416				-3.9	*chpD*	transcriptional regulator
PA0490				-3.4		hypothetical protein
PA0509	-3.0	-6.6			*nirN*	cytochrome C
PA0510				-7.1	*nirE*	uroporphyrin-III C-methyltransferase
PA0511				-4.1	*nirJ*	heme d1 biosynthesis protein NirJ
PA0513	-3.0	-4.7			*nirG*	heme d1 biosynthesis protein NirG
PA0563	-3.6	-3.2				conserved hypothetical protein
PA0567				-4.2		putative stress induced hydrophobic peptide
PA0572			-3.3			immunomodulating metalloprotease
PA0579				-2.9	*rpsU*	30S ribosomal protein S21
PA0593				-3.8	*pdxA*	4-hydroxythreonine-4-phosphate dehydrogenase
PA0612				-6.7	*ptrB*	repressor PtrB
PA0613				-5.8		hypothetical protein
PA0614				-12.4		pyocin R2, holin
PA0615				-6.9		putative phage protein
PA0616				-9.3		putative baseplate assembly protein
PA0617				-13.2		bacteriophage protein
PA0618				-12.4		bacteriophage protein
PA0619				-11.9		bacteriophage protein
PA0620				-12.1		bacteriophage protein
PA0621				-9.7		putative prophage tail fimber assembly protein
PA0622				-11.4		bacteriophage protein
PA0623				-11.4		bacteriophage protein
PA0624				-13.1		hypothetical protein
PA0625				-14.4		putative tail length determinator protein
PA0626				-12.2		putative pyocin R2, tail formation protein gpU
PA0627				-15.6		phage P2 tail gpX-like protein
PA0628				-14.2		putative phage late control gene D protein
PA0629				-15.2		putative phage encoded lysozyme

PA0630				-8.3		hypothetical protein
PA0631				-14.1		hypothetical protein
PA0633				-10.8		hypothetical protein
PA0634				-10.0		hypothetical protein
PA0635				-11.6		putative phage protein
PA0636				-13.6		putative phage tail-like protein
PA0637				-13.1		phage minor tail protein
PA0638				-13.2		bacteriophage protein
PA0639				-12.6		hypothetical protein
PA0640				-13.8		bacteriophage protein
PA0641				-11.3		bacteriophage protein
PA0643				-9.9		putative phage tail fiber protein
PA0644				-9.2		hypothetical protein
PA0645				-7.2		hypothetical protein
PA0646				-7.0		putative phage-like protein
PA0647				-6.9		
PA0648				-5.9		hypothetical protein
PA0654				-2.9	*speD*	S-adenosylmethionine decarboxylase
PA0713	-3.9	-3.0				hypothetical protein
PA0736a				-9.5		hypothetical protein
PA0762				-3.0	*algU*	RNA polymerase sigma factor AlgU
PA0764				-2.9	*mucB*	sigma factor AlgU regulator MucB
PA0807				-13.6	*ampDh3*	protein AmpDh3
PA0808				-2.8		hypothetical protein
PA0809				-4.5		divalent metal cation transporter MntH
PA0812				-3.1		hypothetical protein
PA0813				-4.2		putative aminotransferase
PA0814				-4.8		Rid1 subfamily protein
PA0843				-2.9	*plcR*	phospholipase C accessory protein PlcR
PA0852			-3.9		*cbpD*	chitin-binding protein CbpD
PA0855			-5.3			hypothetical protein
PA0908				-5.0	*alpB*	hypothetical protein
PA0909				-4.7	*alpC*	hypothetical protein
PA0910				-4.4	*alpD*	hypothetical protein
PA0911				-4.5	*alpE*	hypothetical protein
PA0912				-4.5		hypothetical protein
PA0919				-2.9		alanyl-phosphatidylglycerol hydrolase
PA0920				-3.5		alanyl-phosphatidylglycerol synthase
PA0938			-3.7		*wzz2*	hypothetical protein
PA0951a	-5.1	-7.5				hypothetical protein
PA0952	-3.2	-4.7				hypothetical protein
PA0985	-10.1	-25.5			*pyoS5*	pyocin S5
PA0996	-25.9	-17.0			*pqsA*	anthranilate--CoA ligase
PA0997	-16.2	-10.9			*pqsB*	hypothetical protein
PA0998	-8.9	-4.6			*pqsC*	hypothetical protein
PA0999			-6.5		*pqsD*	2-heptyl-4(1H)-quinolone synthase PqsD
PA1000			-8.0		*pqsE*	thioesterase PqsE
PA1001			-5.1		*phnA*	anthranilate synthase component I
PA1077				-14.0	*flgB*	flagellar basal-body rod protein FlgB
PA1078				-11.4	*flgC*	flagellar basal body rod protein FlgC
PA1079				-4.5	*flgD*	flagellar basal body rod modification protein
PA1080				-9.0	*flgE*	flagellar hook protein FlgE
PA1081				-29.8	*flgF*	flagellar basal body rod protein FlgF
PA1082				-21.7	*flgG*	flagellar basal body rod protein FlgG
PA1083				-10.0	*flgH*	flagellar basal body L-ring protein
PA1084				-16.2	*flgI*	flagellar basal body P-ring protein
PA1085				-10.9	*flgJ*	peptidoglycan hydrolase FlgJ
PA1086				-5.8	*flgK*	flagellar hook-associated protein FlgK
PA1087				-5.3	*flgL*	flagellar hook-associated protein FlgL
PA1088				-5.4		putative methyltransferase
PA1089	-3.2	-6.3				hypothetical protein
PA1090	-3.6	-7.2				putative sugar nucleotidyltransferase
PA1091	-3.2	-5.2			*fgtA*	flagellar glycosyl transferase FgtA
PA1092	-33.3	-52.7			*fliC*	B-type flagellin
PA1093	-13.1	-12.7			*flaG*	flagellar protein FlaG
PA1094	-3.3	-7.2			*fliD*	B-type flagellar hook-associated protein
PA1095	-8.0	-10.5			*fliS*	B-type flagellar protein FliS
PA1096	-10.2	-23.2			*fliT*	flagellar assembly protein FliT
PA1098				-3.6	*fleS*	two-component sensor
PA1099	-2.9	-10.6			*fleR*	two-component response regulator
PA1100				-73.5	*fliE*	flagellar hook-basal body complex protein FliE
PA1101	-3.1	-12.9			*fliF*	flagellar MS-ring protein
PA1103	-6.2	-5.3			*fliH*	flagellar assembly protein FliH
PA1104	-8.2	-5.3			*fliI*	flagellum-specific ATP synthase
PA1107			-3.2		*roeA*	hypothetical protein
PA1114				-2.8		hypothetical protein
PA1119	-3.4	-3.0			*yfiB*	hypothetical protein

PA1123	-5.8	-4.5				hypothetical protein
PA1131			-3.5			major facilitator superfamily transporter
PA1132	-7.1	-7.6				hypothetical protein
PA1150				-4.5	*pys2*	pyocin-S2
PA1168				-7.2		hypothetical protein
PA1172			-4.3		*napC*	cytochrome C protein NapC
PA1173			-3.6		*napB*	cytochrome C protein NapB
PA1174			-2.9		*napA*	nitrate reductase catalytic subunit
PA1175			-3.0		*napD*	nitrate reductase biosynthesis protein NapD
PA1202			-3.2			hydrolase
PA1245			-4.2			hypothetical protein
PA1246			-4.4		*aprD*	alkaline protease secretion ATP-binding protein AprD
PA1247			-4.0		*aprE*	alkaline protease secretion protein AprE
PA1248			-5.0		*aprF*	alkaline protease secretion protein AprF
PA1249			-5.4		*aprA*	alkaline metalloproteinase
PA1250			-4.2		*aprI*	alkaline proteinase inhibitor AprI
PA1251			-4.3			chemotaxis transducer
PA1275				-3.1	*cobD*	cobalamin biosynthesis protein CobD
PA1276				-3.0	*cobC*	threonine-phosphate decarboxylase
PA1277				-2.9	*cobQ*	cobyric acid synthase
PA1279				-3.8	*cobU*	nicotinate-nucleotide--dimethylbenzimidazole phosphoribosyltransferase
PA1323				-5.2		hypothetical protein
PA1324				-4.0		hypothetical protein
PA1331	-4.5	-3.9			*yegH*	putative membrane protein, TerC family
PA1370	-4.1	-3.6				hypothetical protein
PA1371	-6.1	-4.5				hypothetical protein
PA1431			-8.7		*rsaL*	regulatory protein RsaL
PA1441				-19.9	*fliK*	putative flagellar hook-length control protein FliK
PA1442				-2.9	*fliL*	flagellar basal body protein FliL
PA1448				-2.9	*fliR*	flagellar biosynthesis protein FliR
PA1449				-3.1	*flhB*	flagellar biosynthesis protein FlhB
PA1450			-2.9			hypothetical protein
PA1452				-8.8	*flhA*	flagellar biosynthesis protein FlhA
PA1458	-2.9	-2.9			*cheA*	two-component sensor
PA1460	-3.3	-3.5			*motC*	flagellar motor protein
PA1461	-3.7	-3.5			*motD*	flagellar motor protein MotD
PA1471				-8.4		hypothetical protein
PA1473	-4.6	-5.2				flagellar protein FhlB-like protein
PA1474	-3.6	-3.4				hypothetical protein
PA1476			-3.3		*ccmB*	heme exporter protein CcmB
PA1478				-3.1	*ccmD*	heme exporter protein CcmD
PA1508			-3.1			hypothetical protein
PA1545	-6.3	-6.8				hypothetical protein
PA1547			-3.3			hypothetical protein
PA1549	-5.8	-4.0			*copA2*	cation-transporting P-type ATPase
PA1550			-4.8			hypothetical protein
PA1551			-3.8			ferredoxin
PA1561			-2.9		*aer*	aerotaxis receptor Aer
PA1592				-64.1		hypothetical protein
PA1656			-8.9		*hsiA2*	T6SS secretion-associated protein
PA1657	-21.2	-5.6			*hsiB2*	T6SS protein TssB2
PA1658	-10.1	-3.1			*hsiC2*	T6SS protein TssC2
PA1659			-10.8		*hsiF2*	T6SS secretion system lysozyme-like protein
PA1660			-5.4		*hsiG2*	T6SS secretion protein TssF2
PA1661	-10.6	-5.3			*hsiH2*	T6SS protein TssG2
PA1662	-5.7	-3.6			*clpV2*	T6SS ATPase, ClpV1 family
PA1663	-7.4	-3.6			*sfa2*	transcriptional regulator
PA1664	-11.3	-6.7			*orfX*	T6SS-associated lipoprotein TagU
PA1665	-14.8	-5.1			*fha2*	T6SS secretion protein Fha2
PA1666			-4.0		*lip2*	T6SS secretion lipoprotein TssJ2
PA1668	-5.8	-4.8			*dotU2*	hypothetical protein
PA1670			-3.2		*stp1*	serine/threonine phosphoprotein phosphatase Stp1
PA1679	-5.8	-6.1				hypothetical protein
PA1744	-4.4	-5.5				hypothetical protein
PA1745			-3.0			hypothetical protein
PA1791			-2.9			hypothetical protein
PA1869			-3.4		*acp1*	acyl carrier protein
PA1871			-3.5		*lasA*	protease LasA
PA1888			-3.5			hypothetical protein
PA1891				-6.0		hypothetical protein
PA1892				-4.4		hypothetical protein
PA1894	-3.5	-5.0				hypothetical protein
PA1899			-4.5		*phzA2*	phenazine biosynthesis protein PhzA
PA1900	-5.3	-3.1			*phzB2*	phenazine biosynthesis protein PhzB
PA1902			-3.5		*phzD2*	phenazine biosynthesis protein PhzD
PA1904			-4.2		*phzF2*	trans-2,3-dihydro-3-hydroxyanthranilate isomerase
PA1913	-24.8	-14.2				hypothetical protein

PA1942	-37.3	-31.7				hypothetical protein
PA1967				-18.4		hypothetical protein
PA1970	-31.3	-37.6				lipoprotein
PA1983				-3.1	*exaB*	cytochrome C550
PA2021				-3.0		hypothetical protein
PA2030			-3.3			hypothetical protein
PA2031			-3.1			hypothetical protein
PA2067	-2.9	-3.1				hydrolase
PA2068	-3.7	-4.6				major facilitator superfamily transporter
PA2069	-2.9	-3.2				carbamoyl transferase
PA2091			-3.5			hypothetical protein
PA2110				-3.6		putative allophanate hydrolase subunit 2
PA2111				-3.9		putative allophanate hydrolase subunit 1
PA2146				-3.9		hypothetical protein
PA2193			-3.9		*hcnA*	hydrogen cyanide synthase subunit HcnA
PA2194			-3.2		*hcnB*	hydrogen cyanide synthase subunit HcnB
PA2195			-3.3		*hcnC*	hydrogen cyanide synthase subunit HcnC
PA2222			-4.6			hypothetical protein
PA2223			-5.9			hypothetical protein
PA2224			-5.7			hypothetical protein
PA2225			-3.8			hypothetical protein
PA2226			-3.6		*qsrO*	hypothetical protein
PA2227			-3.0		*vqsM*	HTH-type transcriptional regulator VqsM
PA2238			-3.0		*pslH*	biofilm formation protein PslH
PA2239			-2.9		*pslI*	biofilm formation protein PslI
PA2240				-4.1	*pslJ*	biofilm formation protein PslJ
PA2241				-3.1	*pslK*	biofilm formation protein PslL
PA2242				-2.9	*pslL*	putative acetyltransferase
PA2289	-3.3	-2.8				hypothetical protein
PA2302	-11.1	-4.2			*ambE*	protein AmbE
PA2303	-8.4	-3.2			*ambD*	protein AmbD
PA2304			-7.3		*ambC*	protein AmbC
PA2305			-6.1		*ambB*	protein AmbB
PA2319			-4.5			transposase
PA2330			-3.1			hypothetical protein
PA2331			-3.9			hypothetical protein
PA2360	-10.8	-4.4			*hsiA3*	T6SS protein
PA2361	-9.9	-4.1			*icmF3*	T6SS protein IcmF
PA2362	-9.7	-4.0			*dotU3*	T6SS protein DotU
PA2363			-5.1		*hsiJ3*	putative T6SS protein
PA2364			-3.2		*lip3*	putative T6SS protein
PA2365			-9.1		*hsiB3*	putative T6SS protein
PA2366	-6.5	-3.5			*hsiC3*	T6SS system uricase
PA2367	-8.4	-4.8			*hcp3*	T6SS system effector
PA2368	-49.8	-14.8			*hsiF3*	T6SS protein
PA2369	-15.1	-9.4			*hsiG3*	T6SS system protein
PA2370	-25.4	-9.8			*hsiH3*	T6SS protein
PA2371	-9.9	-5.1			*clpV3*	ClpA/B-type protease
PA2373	-5.4	-2.9			*vgrG3*	type IV secretion protein Rhs
PA2374	-5.9	-3.1			*tseF*	hypothetical protein
PA2381			-3.5			hypothetical protein
PA2384	-11.1	-34.5				ferric uptake regulator family protein
PA2386				-11.8	*pvdA*	L-ornithine N5-oxygenase
PA2390			-2.9		*pvdT*	pyoverdine biosynthesis protein PvdT
PA2391	-3.1	-6.7			*opmQ*	hypothetical protein
PA2398	-3.9	-4.8			*fpvA*	ferripyoverdine receptor
PA2400				-10.6	*pvdJ*	pyoverdine biosynthesis protein PvdJ
PA2403	-5.0	-10.4			*fpvG*	iron-regulated membrane protein
PA2404	-5.4	-14.1			*fpvH*	thiamine pyrophosphate-requiring enzyme-like protein
PA2405	-5.4	-11.4			*fpvJ*	hypothetical protein
PA2406	-4.6	-13.9			*fpvK*	hypothetical protein
PA2407	-6.9	-13.8			*fpvC*	adhesion protein
PA2408	-15.8	-42.3			*fpvD*	ABC transporter ATP-binding protein
PA2409	-8.3	-11.2			*fpvE*	ABC transporter permease
PA2410	-3.3	-4.8			*fpvF*	putative ABC transporter substrate-binding protein
PA2411				-24.2		thioesterase
PA2412	-3.2	-57.1				MbtH domain-containing protein
PA2413				-16.4	*pvdH*	diaminobutyrate--2-oxoglutarate aminotransferase
PA2423			-3.6			hypothetical protein
PA2425	-4.4	-11.4			*pvdG*	pyoverdine biosynthesis protein PvdG
PA2426			-3.4		*pvdS*	extracytoplasmic-function sigma-70 factor
PA2433				-8.7		hypothetical protein
PA2442				-4.0	*gcvT2*	glycine cleavage system protein T2
PA2462	-4.4	-3.2				filamentous hemagglutinin-like protein
PA2463	-11.4	-5.2				putative hemolysin activation/secretion protein
PA2475			-3.2			cytochrome P450
PA2485	-7.4	-20.3				hypothetical protein

PA2486	-13.3	-24.9			*ptrC*	Pseudomonas type III repressor gene C, PtrC
PA2491	-3.7	-3.3			*mexS*	oxidoreductase
PA2493	-132.4	-66.9			*mexE*	resistance-nodulation-cell division (RND) multidrug efflux membrane fusion protein MexE
PA2494	-22.2	-16.7			*mexF*	resistance-nodulation-cell division (RND) multidrug efflux transporter MexF
PA2495	-38.2	-30.8			*oprN*	multidrug efflux outer membrane protein OprN
PA2501				-3.3		hypothetical protein
PA2514			-3.4		*antC*	anthranilate dioxygenase reductase
PA2515			-4.8		*xylL*	1,6-dihydroxycyclohexa-2,4-diene-1-carboxylate dehydrogenase
PA2537			-3.0			acyltransferase
PA2538	-3.6	-3.0				hypothetical protein
PA2539	-4.0	-3.0			*ynbD*	Ser/Thr and Tyr protein phosphatase (dual specificity)
PA2559a				-2.8	*srfA*	hypothetical protein
PA2566			-3.8			putative pyridine nucleotide-disulfide oxidoreductase
PA2567				-3.3		putative diguanylate phosphodiesterase
PA2569				-3.7		hypothetical protein
PA2570	-4.1	-3.1			*lecA*	PA-I galactophilic lectin
PA2572			-2.9			two-component response regulator
PA2579			-2.9		*kynA*	tryptophan 2,3-dioxygenase
PA2587			-3.1		*pqsH*	2-heptyl-3-hydroxy-4(1H)-quinolone synthase
PA2588			-3.5			transcriptional regulator
PA2591			-2.9		*vqsR*	transcriptional regulator
PA2592			-4.5			spermidine/putrescine-binding protein
PA2593			-3.6		*qteE*	quorum threshold expression protein QteE
PA2619				-5.7	*infA*	translation initiation factor IF-1
PA2622			-2.9		*cspD*	cold-shock protein CspD
PA2652	-5.3	-5.5				methyl-accepting chemotaxis protein
PA2654	-4.4	-11.0			*tlpQ*	methyl-accepting chemotaxis sensory transducer
PA2703			-3.9			hypothetical protein
PA2730			-3.7			hypothetical protein
PA2754				-3.7		hypothetical protein
PA2755a			-3.1			hypothetical protein
PA2759	-12.9	-10.5				hypothetical protein
PA2774			-3.1		*tse4*	hypothetical protein
PA2780				-2.9	*bswR*	bacterial swarming regulator BswR
PA2781				-2.8		hypothetical protein
PA2787	-4.2	-3.8			*cpg2*	glutamate carboxypeptidase
PA2788	-11.7	-8.5				chemotaxis transducer
PA2792	-6.6	-2.9				hypothetical protein
PA2811	-3.1	-3.5			*yadH*	ABC transporter permease
PA2867	-6.5	-8.9				methyl-accepting chemotaxis sensory transducer
PA2874	-4.2	-3.9				hypothetical protein
PA2895				-3.0	*sbrR*	hypothetical protein
PA2909				-2.9		cobalt-precorrin-6x reductase
PA2916				-3.2		hypothetical protein
PA2939			-5.4			aminopeptidase
PA2955				-3.0		putative lipoprotein
PA2964				-3.0	*pabC*	4-amino-4-deoxychorismate lyase
PA3021			-3.9			hypothetical protein
PA3069				-4.5		putative lipoprotein
PA3095	-3.0	-4.0			*xcpZ*	type II secretion system protein M
PA3105	-3.5	-2.8			*xcpQ*	type II secretion system protein D
PA3143			-2.8			transposase
PA3145	-3.1	-2.9			*wbpL*	glycosyltransferase WbpL
PA3146	-4.4	-4.2			*wbpK*	NAD-dependent epimerase/dehydratase
PA3147	-4.5	-4.5			*wbpJ*	glycosyl transferase WbpJ
PA3148	-5.8	-6.9			*wbpI*	UDP-2,3-diacetamido-2,3-dideoxy-D-glucuronate 2-epimeras
PA3149	-6.9	-6.7			*wbpH*	glycosyltransferase WbpH
PA3150	-4.3	-4.9			*wbpG*	LPS biosynthesis protein WbpG
PA3151	-4.8	-5.2			*hisF2*	imidazole glycerol phosphate synthase subunit HisF
PA3152	-5.3	-5.0			*hisH2*	imidazole glycerol phosphate synthase subunit HisH
PA3153	-7.2	-5.5			*wzx*	O-antigen translocase
PA3154	-4.7	-3.9			*wzy*	B-band O-antigen polymerase
PA3155	-3.5	-3.1			*wbpE*	UDP-2-acetamido-2-deoxy-3-oxo-D-glucuronate aminotransferase
PA3156			-2.9		*wbpD*	UDP-2-acetamido-3-amino-2, 3-dideoxy-D-glucuronate N-acetyltransferase
PA3186				-12.7	*oprB*	glucose/carbohydrate outer membrane porin OprB precursor
PA3187				-17.2	*gltK*	ABC transporter ATP-binding protein
PA3188				-16.1	*gltG*	sugar ABC transporter permease
PA3189				-16.8	*gltF*	sugar ABC transporter permease
PA3190				-12.8	*gltB*	sugar ABC transporter substrate-binding protein
PA3229	-88.5	-69.2				hypothetical protein with upstream mexT binding site
PA3230	-4.8	-4.9				hypothetical protein
PA3274				-4.6		hypothetical protein
PA3278				-3.0		hypothetical protein
PA3291	-7.8	-6.1			*tli1*	hypothetical protein

PA3292	-7.5	-4.7				hypothetical protein
PA3293			-3.2			hypothetical protein
PA3294			-4.0		*vgrG4a*	T6SS secretion protein Rhs
PA3309				-2.8	*uspK*	hypothetical protein
PA3311			-3.3		*nbdA*	signaling protein/diguanylate cyclase/phosphodiesterase
PA3325			-2.9			putative hydrolase
PA3326			-3.1		*clpP2*	ATP-dependent Clp protease proteolytic subunit
PA3327			-4.9			non-ribosomal peptide synthetase
PA3328			-7.8			FAD-dependent monooxygenase
PA3329			-6.8			hypothetical protein
PA3330			-6.7			short-chain dehydrogenase
PA3331			-4.8			cytochrome P450
PA3332			-7.8			hypothetical protein
PA3333			-6.2		*fabH2*	3-oxoacyl-ACP synthase
PA3334			-7.4		*acp3*	acyl carrier protein
PA3335			-6.4			hypothetical protein
PA3336			-5.4			major facilitator superfamily transporter
PA3341			-4.1			transcriptional regulator
PA3346			-2.9			two-component response regulator
PA3349	-3.5	-3.3				chemotaxis protein
PA3350	-2.9	-6.8			*flgA*	flagellar basal body P-ring biosynthesis protein FlgA
PA3351	-4.7	-4.5			*flgM*	protein FlgM
PA3352	-3.5	-2.9			*flgN*	flagellar biosynthesis protein FlgN
PA3353	-4.5	-3.2			*flgZ*	type IV pilus assembly protein, PilZ family
PA3361			-3.8		*lecB*	fucose-binding lectin PA-IIL
PA3385	-12.6	-8.3			*amrZ*	alginate and motility regulator Z
PA3386	-4.1	-3.8				hypothetical protein
PA3403a				-13.3		hypothetical protein
PA3410				-3.2	*hasI*	ECF subfamily sigma-70 factor
PA3411				-3.0		hypothetical protein
PA3415			-3.2			branched-chain alpha-keto acid dehydrogenase subunit E2
PA3472				-4.2		NLP/P60 protein
PA3477			-4.8		*rhlR*	transcriptional regulator RhlR
PA3479			-3.1		*rhlA*	rhamnosyltransferase subunit A
PA3484			-3.4		*tse3*	hypothetical protein
PA3485	-5.9	-4.8			*tsi3*	hypothetical protein
PA3486			-3.2		*vgrG4b*	Rhs element Vgr protein
PA3487			-3.2		*pldA*	phospholipase D
PA3520			-5.0			putative periplasmic substrate binding protein
PA3526	-7.3	-8.4			*motY*	putative exported flagellar protein
PA3535			-3.1			serine protease
PA3569				-20.8	*mmsB*	3-hydroxyisobutyrate dehydrogenase
PA3570				-12.8	*mmsA*	methylmalonate-semialdehyde dehydrogenase
PA3621				-2.9	*fdxA*	ferredoxin I
PA3642				-3.0	*rnhB*	ribonuclease HII
PA3662			-3.8			hypothetical protein
PA3691				-3.6		putative chromosome segregation ATPase
PA3710				-2.8		GMC-type oxidoreductase
PA3721	-2.8	-3.1			*nalC*	transcriptional regulator
PA3722	-19.4	-15.5				hypothetical protein
PA3724			-3.7		*lasB*	elastase LasB
PA3727			-4.5			nuclease-like protein
PA3733a				-4.1		hypothetical protein
PA3740	-3.9	-3.7				hypothetical protein
PA3747				-2.8		cytochrome c assembly protein
PA3762	-3.0	-4.2				hypothetical protein
PA3784			-5.3			hypothetical protein
PA3785			-5.2			hypothetical protein
PA3789			-3.8			peptidase
PA3790			-3.6		*oprC*	copper transport outer membrane porin OprC
PA3791			-3.9			hypothetical protein
PA3819				-20.3		hypothetical protein
PA3865a				-3.6		hypothetical protein
PA3866	-3.7	-12.7				pyocin protein
PA3900				-3.1	*fecR*	transmembrane sensor
PA3904			-7.9			hypothetical protein
PA3905	-32.6	-6.8			*tecT*	type VI effector chaperone for Tox-Rease, TecT
PA3906	-24.7	-6.4				co-chaperone, co-TecT
PA3907	-23.1	-8.2			*tseT*	TOX-REase-5 domain-containing effector, TseT
PA3908	-28.4	-8.7			*tsiT*	immunity protein, TsiT
PA3911	-2.9	-4.1				putative lipid carrier protein
PA3930			-3.2		*cioA*	cyanide insensitive terminal oxidase
PA3982			-3.4			metalloprotease
PA3983	-8.8	-7.4				magnesium and cobalt efflux protein
PA4045				-3.2	*yadT*	hypothetical protein
PA4112	-3.1	-3.3				sensor/response regulator hybrid protein
PA4117			-3.0		*bphP*	phytochrome BphP

PA4127			-3.4		*hpcG*	2-oxo-hepta-3-ene-1,7-dioic acid hydratase
PA4128			-4.7		*hpcH*	hypothetical protein
PA4129			-4.3			hypothetical protein
PA4130			-3.4			sulfite/nitrite reductase
PA4131			-3.3			iron-sulfur protein
PA4132			-3.7		*mpaR*	transcriptional regulator, GntR family domain / Aspartate aminotransferase
PA4133			-8.8			cbb3-type cytochrome C oxidase subunit I
PA4134			-7.6			hypothetical protein
PA4136			-3.1			major facilitator superfamily transporter
PA4139				-5.8		hypothetical protein
PA4141			-3.3			hypothetical protein
PA4175			-3.4		*piv*	endopeptidase IV
PA4210			-5.3		*phzA1*	phenazine biosynthesis protein
PA4211			-7.4		*phzB1*	phenazine biosynthesis protein
PA4212			-7.8		*phzC1*	phenazine biosynthesis protein PhzC
PA4217			-3.7		*phzS*	hypothetical protein
PA4218				-3.7	*ampP*	transporter
PA4219				-3.2	*ampO*	hypothetical protein
PA4224				-3.4	*pchG*	pyochelin biosynthetic protein PchG
PA4225				-3.9	*pchF*	pyochelin synthetase
PA4226				-3.6	*pchE*	dihydroaeruginoic acid synthetase
PA4229				-4.0	*pchC*	pyochelin biosynthetic protein PchC
PA4294			-3.2			putative pilus assembly protein
PA4296	-6.5	-4.0			*pprB*	two-component response regulator PprB
PA4297			-3.0		*tadG*	hypothetical protein
PA4298			-4.1			hypothetical protein
PA4299			-4.7		*tadD*	type II secretion system protein TadD
PA4300			-5.6		*tadC*	type II secretion system protein TadC
PA4301			-3.6		*tadB*	type II secretion system protein TadB
PA4302			-6.1		*tadA*	ATPase TadA
PA4304			-4.4		*rcpA*	type II/III secretion system protein
PA4305			-5.2		*rcpC*	hypothetical protein
PA4306	-25.6	-6.4			*flp*	type IVb pilin Flp
PA4307			-7.8		*pctC*	chemotactic transducer PctC
PA4310	-7.7	-6.4			*pctB*	chemotactic transducer PctB
PA4318	-3.6	-3.0				hypothetical protein
PA4319	-5.6	-4.4				integral membrane protein
PA4320	-3.7	-3.4				hypothetical protein
PA4321	-5.2	-4.8				hypothetical protein
PA4322	-3.6	-3.4				ATPase
PA4323	-2.9	-3.9				putative transmembrane protein
PA4324	-3.0	-4.3				type IV pilus assembly PilZ
PA4326	-8.9	-9.7				putative lipoprotein
PA4331			-2.8			probable ferredoxin reductase
PA4332			-4.0		*sadC*	diguanylate cyclase
PA4346				-2.8		hypothetical protein
PA4354	-7.2	-4.0				putative ArsR family regulatory protein
PA4355	-6.2	-4.0			*pyeM*	major facilitator superfamily transporter
PA4356			-4.3		*xenB*	xenobiotic reductase
PA4357				-4.5	*yhgG*	hypothetical protein
PA4358				-3.1	*feoB*	ferrous iron transporter B
PA4359	-3.4	-3.0			*feoA*	ferrous iron transporter A
PA4455				-2.9	*yrbE*	ABC transporter permease
PA4479				-2.9	*mreD*	rod shape-determining protein MreD
PA4487	-4.4	-3.8			*magF*	hypothetical protein
PA4489	-4.9	-3.7			*magD*	alpha-2-macroglobulin family protein
PA4490	-4.3	-3.2			*magC*	hypothetical protein
PA4491	-4.3	-3.2			*magB*	hypothetical protein
PA4525	-49.0	-54.7			*pilA*	type 4 fimbrial protein PilA
PA4591	-3.1	-3.0				secretion protein HlyD family protein
PA4592	-3.4	-4.6			*opmF*	probable outer membrane protein precursor
PA4593			-3.4			ABC transporter permease
PA4601	-3.8	-3.0			*morA*	motility regulator
PA4623	-42.2	-46.5				hypothetical protein with upstream mexT binding site
PA4633	-4.1	-3.6				chemotaxis transducer
PA4637a	-4.3	-4.2				hypothetical protein
PA4648			-5.8		*cupE1*	hypothetical protein
PA4651			-3.3			pili assembly chaperone
PA4683				-4.6		hypothetical protein
PA4738			-3.4			hypothetical protein
PA4739			-3.3			transport-associated protein
PA4778			-4.7		*cueR*	protein CueR
PA4843	-10.4	-8.9			*gcbA*	two-component response regulator
PA4867			-2.8		*ureB*	urease subunit beta
PA4876				-3.7	*osmE*	osmE family transcriptional regulator
PA4880				-4.4		bacterioferritin
PA4881	-21.2	-20.3				hypothetical protein

PA4922				-3.7	*azu*	azurin
PA4929			-3.2			diguanylate cyclase
PA4997				-4.3	*msbA*	transporter MsbA
PA5058			-2.9		*phaC2*	poly(3-hydroxyalkanoic acid) synthase
PA5059	-4.2	-3.9			*phaD*	transcriptional regulator
PA5071			-3.0			16S ribosomal RNA methyltransferase RsmE
PA5072				-3.1	*mcpK*	chemotaxis transducer
PA5088			-3.4		*tli5b3*	T6SS lipase immunity protein, Tli5b3
PA5113			-4.4			hypothetical protein
PA5178				-4.3		LysM domain/BON superfamily protein
PA5182				-4.3		hypothetical protein
PA5212				-11.5		ribonucleotide reductase subunit alpha
PA5230	-3.3	-2.9			*yhhJ*	ABC transporter permease
PA5231				-2.9	*yhiH*	ABC transporter ATP-binding protein/permease
PA5267			-5.0		*hcpB*	secreted protein Hcp
PA5339				-3.8		hypothetical protein
PA5352			-5.3		*glcG*	hypothetical protein
PA5353	-3.3	-3.1			*glcF*	glycolate oxidase iron-sulfur subunit
PA5354	-3.3	-4.2			*glcE*	glycolate oxidase FAD binding subunit
PA5355			-3.3		*glcD*	glycolate oxidase subunit GlcD
PA5362	-3.9	-3.6				hypothetical protein
PA5421				-5.9	*fdhA*	glutathione-independent formaldehyde dehydrogenase
PA5424				-4.2		transglycosylase-associated protein
PA5475				-5.1		putative acetyltransferase
PA5526				-7.5		putative lipoprotein

[a] Differential gene expression of Populations E and D grown with added AHLs, day 160 vs. day 5.

[b] Locus tag and gene name from pseudomonas.com (1).
