# Supplementary material for: Evolution of the Quorum Sensing Regulon in Cooperating Populations of Pseudomonas aeruginosa
Source: mBio. 2022 Feb 22;13(1):e00161-22. doi: 10.1128/mbio.00161-22 (PMC8863103; doi:10.1128/mbio.00161-22)
Supplement: TABLE S3 [file mbio.00161-22-st003.pdf]

Supplementary Table 3. Genes with greater expression at day 160 in CAB-evolved Populations D and E, as compared to day 5.

	Fold Change[a]					
	Day 160 lineages					
Locus tag[b]	Pop D	Pop E	D only	E only	Gene name[b]	Description
PA0044	5.1	4.1			*exoT*	exoenzyme T
PA0113			3.3			protoheme IX farnesyltransferase
PA0115			3.8		*elaA*	putative acetyltransferase
PA0117	2.9	3.5				probable short chain dehydrogenase
PA0118				4.5		putative DSBA oxidoreductase
PA0119			3.4		*dctA*	C4-dicarboxylate transporter DctA
PA0120	3.8	4.4				probable transcriptional regulator
PA0121	4.6	4.5				putative transcriptional regulator
PA0127	4.7	4.5				putative lipoprotein
PA0128			2.9		*phnA*	alkylphosphonate utilization protein PhnA
PA0129			3.5		*bauD*	gamma-aminobutyrate permease
PA0130	4.2	4.5			*bauC*	3-Oxopropanoate dehydrogenase
PA0131	5.4	5.3			*bauB*	cupin 2 protein
PA0132			3.6		*bauA*	beta alanine--pyruvate transaminase
PA0133	3.5	3.9			*bauR*	HTH-type transcriptional activator BauR
PA0134			3.9		*gda2*	guanine deaminase
PA0135	4.6	3.4				hypothetical protein
PA0136	4.1	3.7				ABC transporter ATP-binding protein
PA0137	4.5	4.0				probable permease of ABC transporter
PA0138	3.8	4.0				probable permease of ABC transporter
PA0140	3.9	3.9			*ahpF*	alkyl hydroperoxide reductase subunit F
PA0141			3.4			putative polyphosphate kinase
PA0142	4.3	4.3				8-oxoguanine deaminase
PA0143	12.1	15.5			*nuh*	nonspecific ribonucleoside hydrolase
PA0144	5.9	5.9				putative nucleoside 2-deoxyribosyltransferase
PA0145	5.9	5.3				putative nucleoside 2-deoxyribosyltransferase
PA0146	5.3	4.9				putative basic membrane protein
PA0147	4.7	5.8				probable oxidoreductase
PA0148	3.9	4.3			*add*	adenine deaminase
PA0151			4.2			TonB-dependent receptor
PA0152			2.9		*pcaQ*	transcriptional regulator PcaQ
PA0153	5.0	4.1			*pcaH*	protocatechuate 3,4-dioxygenase, beta subunit
PA0154	3.8	3.5			*pcaG*	protocatechuate 3,4-dioxygenase, alpha subunit
PA0159	3.1	3.5				probable transcriptional regulator
PA0160	2.8	3.6				hypothetical protein
PA0162	4.6	11.0			*opdC*	histidine porin OpdC
PA0164	4.6	2.9				probable gamma-glutamyltranspeptidase
PA0165	16.7	11.5				nucleoside-binding outer membrane protein-like protein
PA0166	4.9	4.3				probable transporter
PA0167	4.6	3.4				probable transcriptional regulator
PA0168			3.1		*yigZ*	putative thymidylate synthase
PA0169			6.4		*siaD*	putative diguanylate cyclase
PA0170			7.9			hypothetical protein
PA0171			6.8			hypothetical protein
PA0172			7.4		*siaA*	hypothetical protein
PA0182	4.6	4.4				3-ketoacyl-ACP reductase
PA0183	4.2	4.0			*atsA*	arylsulfatase
PA0184	8.2	6.4			*atsC*	ABC transporter ATP-binding protein
PA0185	4.9	3.8			*atsB*	probable permease of ABC transporter
PA0186	4.3	3.9			*atsR*	probable binding protein component of ABC transporter
PA0187	3.8	3.6				putative gamma-butyrobetaine dioxygenase
PA0188	4.7	4.6				putative transporter
PA0189	3.8	3.1			*opdI*	probable porin
PA0190	4.7	4.2				probable acid phosphatase
PA0191	3.6	3.4				probable porin
PA0192	4.4	5.0				probable TonB-dependent receptor
PA0193	6.7	5.8				putative taurine dioxygenase
PA0194				10.3		taurine dioxygenase
PA0195.1				4.5	*pntAB*	NAD(P) transhydrogenase subunit alpha
PA0202				3.6		amidase
PA0203				7.1		ABC transporter
PA0204				4.2		ABC transporter permease
PA0205				6.5		ABC transporter permease
PA0206				4.3		spermidine/putrescine ABC transporter ATP-binding protein
PA0207				3.7		transcriptional regulator

PA0208				14.5	*mdcA*	malonate decarboxylase subunit alpha
PA0215				3.7	*madL*	malonate transporter MadL
PA0216				3.6	*madM*	malonate transporter MadM
PA0217				4.6	*mdcR*	transcriptional regulator
PA0218				6.5		transcriptional regulator
PA0219				4.7		aldehyde dehydrogenase
PA0220				4.5		amino acid APC family transporter
PA0221				7.5		aminotransferase
PA0222				6.4		putative binding protein component of ABC transporter
PA0223				3.7		dihydrodipicolinate synthetase
PA0224				5.6		class II aldolase/adducin domain-containing protein
PA0225				4.4		transcriptional regulator
PA0226				7.0		CoA transferase subunit A
PA0227				4.2		CoA transferase subunit B
PA0228				3.5	*pcaF*	beta-ketoadipyl CoA thiolase
PA0229				4.5	*pcaT*	dicarboxylic acid transporter PcaT
PA0230				3.6	*pcaB*	3-carboxy-cis,cis-muconate cycloisomerase
PA0231				5.7	*pcaD*	beta-ketoadipate enol-lactone hydrolase
PA0232				4.9	*pcaC*	gamma-carboxymuconolactone decarboxylase
PA0233				4.1		transcriptional regulator
PA0234	3.9	10.7				hypothetical protein
PA0235				11.0	*pcaK*	4-hydroxybenzoate transporter PcaK
PA0237				4.2		oxidoreductase
PA0238				3.0		putative xylose isomerase
PA0239				3.4		putative permease
PA0240				4.2	*opdF*	porin
PA0241				3.6		major facilitator superfamily transporter
PA0242				3.7		putative 4-hydroxyphenylpyruvate dioxygenase
PA0246				3.9		major facilitator superfamily transporter
PA0247				4.5	*pobA*	p-hydroxybenzoate hydroxylase
PA0248				3.3	*pobR*	transcriptional regulator PobR
PA0249				4.4		acetyltransferase
PA0250				3.6		signal-transduction protein
PA0251				3.6		hypothetical protein
PA0255				3.9		putative transmembrane protein
PA0257				3.0		integrase catalytic subunit
PA0265	3.8	4.5			*davD*	glutaric semialdehyde dehydrogenase DavD
PA0266	3.3	4.2			*davT*	delta-aminovalerate aminotransferase
PA0291				4.1	*oprE*	anaerobically-induced outer membrane porin OprE
PA0296				3.1	*spuI*	glutamine synthetase
PA0298	3.9	4.2			*spuB*	glutamine synthetase
PA0299	3.7	4.5			*spuC*	aminotransferase
PA0301	2.9	3.8			*spuE*	spermidine ABC transporter substrate-binding protein SpuE
PA0302	3.6	5.0			*spuF*	polyamine transporter PotG
PA0321	7.0	3.3				acetylpolyamine amidohydrolase
PA0322	4.7	3.8				probable transporter
PA0326			4.2			ABC transporter ATP-binding protein
PA0432				3.6	*sahH*	adenosylhomocysteinase
PA0441				3.5	*dht*	D-hydantoinase/dihydropyrimidinase
PA0446				3.3		putative lipid metabolism-related protein
PA0447	4.6	5.8			*gcdH*	glutaryl-CoA dehydrogenase
PA0474			17.0			esterase
PA0475			2.9			transcriptional regulator
PA0476	4.7	3.1				probable permease
PA0482	3.0	7.3			*glcB*	malate synthase G
PA0492			7.8		*ycsF*	hypothetical protein
PA0493			9.4			probable biotin-requiring enzyme
PA0494			4.3			acetyl-CoA carboxylase biotin carboxylase subunit
PA0495			2.9			putative allophanate hydrolase subunit 1
PA0496			4.4		*ybgK*	putative hydrolase
PA0500				3.7	*bioB*	biotin synthase
PA0501				3.1	*bioF*	8-amino-7-oxononanoate synthase
PA0534			3.4		*pauB1*	FAD-dependent oxidoreductase
PA0603			5.0		*agtA*	ABC transporter ATP-binding protein
PA0604	9.5	2.9			*agtB*	Polyamine ABC transporter, periplasmic polyamine-binding protein
PA0605			3.0		*agtC*	ABC transporter permease
PA0650				3.0	*trpD*	anthranilate phosphoribosyltransferase
PA0758	5.0	3.5				putative signal transduction protein
PA0779				3.2	*asrA*	ATP-dependent protease AsrA
PA0792			3.7		*prpD*	2-methylcitrate dehydratase
PA0793			4.3			putative AcnD-accessory protein PrpF

PA0794			7.3			aconitate hydratase
PA0795	7.6	3.2			*prpC*	methylcitrate synthase
PA0796	3.3	2.9			*prpB*	2-methylisocitrate lyase
PA0841				3.4		hypothetical protein
PA0865	3.4	3.7			*hpd*	4-hydroxyphenylpyruvate dioxygenase
PA0866			3.8		*aroP2*	aromatic amino acid transporter AroP
PA0887	8.4	29.8			*acsA*	acetyl-coenzyme A synthetase
PA0913	6.2	6.7			*mgtE*	Mg (2+) transporter MgtE
PA0958	4.2	3.2			*oprD*	basic amino acid outer membrane porin OprD precursor
PA1019a			3.4			thioesterase
PA1026				2.8		transcription elongation factor
PA1027				4.3	*amaB*	delta1-Piperideine-6-carboxylate dehydrogenase
PA1051			5.9			putative gluconate transporter
PA1074	3.2	3.7			*braC*	branched-chain amino acid transport protein BraC
PA1140	4.3	10.6			*ylbA*	conserved hypothetical protein
PA1169			3.5			arachidonate 15-lipoxygenase
PA1183	2.8	28.7			*dctA*	C4-dicarboxylate transport protein
PA1190	4.4	4.6			*yohC*	conserved hypothetical protein
PA1203				3.0		redox protein, regulator of disulfide bond formation
PA1205				2.9		quercetin 2,3-dioxygenase
PA1229				3.8	*yeaM*	transcriptional regulator
PA1260			3.2		*lhpP*	amino acid ABC transporter substrate-binding protein
PA1297	3.0	3.6				metal transporter
PA1298				2.9		putative transcriptional repressor protein
PA1325	5.5	8.7			*yybH*	hypothetical protein
PA1326	4.2	7.6			*ilvA2*	threonine dehydratase, biosynthetic
PA1409	9.2	5.3			*aphA*	acetylpolyamine aminohydrolase
PA1410	8.1	4.7			*potF1*	spermidine/putrescine-binding protein
PA1418				3.0		sodium:solute symport protein
PA1421				3.4	*gbuA*	guanidinobutyrase
PA1433				3.3		diguanylate cyclase/phosphodiesterase
PA1498	4.7	18.5			*pykF*	pyruvate kinase
PA1499	9.0	51.3				putative hydroxypyruvate reductase
PA1500	10.4	62.5				probable oxidoreductase
PA1501	4.1	21.1				hydroxypyruvate isomerase
PA1502	10.5	47.9			*gcl*	glyoxylate carboligase
PA1503	5.1	51.5				hypothetical protein
PA1507	7.5	15.3				probable transporter
PA1513				8.1		hypothetical protein
PA1514				9.3	*ybbT*	ureidoglycolate hydrolase YbbT
PA1515	3.7	12.6			*alc*	allantoicase
PA1516	6.6	22.7				putative uricase
PA1517	6.3	19.0				chitin/polysaccharide deacetylase family protein
PA1518	4.9	16.5				putative purine catabolism-like protein OR hydroxyisourate hydrolase
PA1519	5.9	56.5				xanthine/uracil permeases family protein
PA1521	3.2	44.4			*gda1*	probable guanine deaminase
PA1522				19.1	*xdhC*	xanthine dehydrogenase accessory factor XdhC
PA1523	5.3	51.8			*xdhB*	xanthine dehydrogenase
PA1524	6.5	75.6			*xdhA*	xanthine dehydrogenase
PA1588				2.8	*sucC*	succinyl-CoA ligase subunit beta
PA1602			4.7			oxidoreductase
PA1606			3.9			putative helicase subunit of the DNA excision repair complex
PA1631				3.6		acyl-CoA dehydrogenase
PA1632			6.7		*kdpF*	potassium-transporting ATPase subunit F
PA1633			8.9		*kdpA*	potassium-transporting ATPase subunit A
PA1634			26.2		*kdpB*	potassium-transporting ATPase subunit B
PA1635			35.4		*kdpC*	potassium-transporting ATPase subunit C
PA1654				3.2		aminotransferase
PA1655				3.0		glutathione S-transferase
PA1677				3.2		isochorismatase hydrolase
PA1692			3.8		*pscS*	translocation protein in type III secretion
PA1694			3.8		*pscQ*	type III secretion system protein
PA1695	4.1	3.7			*pscP*	translocation protein in type III secretion
PA1696	7.0	3.9			*pscO*	translocation protein in type III secretion
PA1697	14.8	4.0			*pscN*	ATP synthase in type III secretion system
PA1698	17.2	6.0			*popN*	type III secretion outer membrane protein PopN precursor
PA1699			4.8		*pcr1*	putative protein in type III secretion
PA1700			3.0		*pcr2*	putative type III secretion protein
PA1701			31.8		*pcr3*	hypothetical protein in type III secretion
PA1702			3.7		*pcr4*	hypothetical protein in type III secretion
PA1705	16.2	10.2			*pcrG*	type III secretion regulator

PA1706	10.0	5.7			*pcrV*	type III secretion protein PcrV
PA1707	10.4	7.6			*pcrH*	regulatory protein PcrH
PA1708	7.8	4.4			*popB*	translocator protein PopB
PA1709	7.9	6.8			*popD*	translocator outer membrane protein PopD
PA1710	5.0	5.0			*exsC*	ExsC, exoenzyme S synthesis protein C precursor
PA1711	5.0	6.2			*exsE*	ExsE is secreted by the type III secretion system and functions as a regulator of ExsC
PA1712	5.3	4.9			*exsB*	exoenzyme S synthesis protein ExsB
PA1719			4.8		*pscF*	type III export protein PscF
PA1742	3.4	3.6			*pauD2*	Glutamine amidotransferase class I
PA1755			3.1			hypothetical protein
PA1761			3.6			hypothetical protein
PA1946	9.8	5.6			*rbsB*	ribose ABC transporter substrate-binding protein
PA1947	10.3	6.3			*rbsA*	ribose transporter RbsA
PA1948	7.4	5.9			*rbsC*	ribose ABC transporter permease
PA1949	6.4	4.3			*rbsR*	ribose operon repressor RbsR
PA1950			3.6		*rbsK*	ribokinase
PA1952			4.1		*fapE*	hypothetical protein
PA1959			3.0		*bacA*	undecaprenyl-diphosphatase
PA1975			4.4			putative methyl-accepting chemotaxis protein
PA1977			4.9			putative transmembrane protein
PA1978	6.0	4.8			*erbR*	response regulator ErbR
PA1979			5.6		*eraS*	sensor kinase EraS
PA1980			7.1		*eraR*	response regulator EraR
PA1983			5.5		*exaB*	cytochrome C550
PA1984	5.4	3.5			*exaC*	NAD+ dependent aldehyde dehydrogenase ExaC
PA1992			3.0		*ercS*	sensor histidine kinase
PA1999	3.4	3.5			*dhcA*	dehydrocarnitine CoA transferase subunit A
PA2000	3.2	4.7			*dhcB*	dehydrocarnitine CoA transferase, subunit B
PA2002	3.4	3.2			*atoE*	short-chain fatty acid transporter family protein
PA2004			3.1			putative citrate transporter
PA2008				3.1	*fahA*	fumarylacetoacetase
PA2040	5.1	7.3			*pauA4*	Glutamylpolyamine synthetase
PA2120				3.1		hypothetical protein
PA2137	2.8	4.7				putative two-component system response regulator protein
PA2166				15.0		hypothetical protein
PA2170			17.0			hypothetical protein
PA2173				3.0		hypothetical protein
PA2191	4.6	2.9			*exoY*	adenylate cyclase
PA2209			3.6			putative tricarboxylate transporter
PA2259				2.9	*ptxS*	transcriptional regulator PtxS
PA2260				3.4		putative xylose isomerase
PA2263				3.2	*kguD*	2-hydroxyacid dehydrogenase
PA2264				2.9		putative gluconate 2-dehydrogenase subunit
PA2322	3.1	3.0			*gntP*	gluconate permease
PA2323	2.8	4.4			*gapN*	probable glyceraldehyde-3-phosphate dehydrogenase
PA2428				3.8		hypothetical protein
PA2508				3.3	*catC*	muconolactone delta-isomerase
PA2523			2.8		*czcR*	two-component response regulator
PA2553			3.2			acyl-CoA thiolase
PA2554			3.2			short-chain dehydrogenase
PA2555			3.1			AMP-binding protein
PA2557			3.2			AMP-binding protein
PA2624				5.8	*idh*	isocitrate dehydrogenase
PA2679				6.8		SAM-dependent methyltransferase
PA2747				7.7		hypothetical protein
PA2776	7.8	8.6			*pauB3*	FAD-dependent oxidoreductase
PA2825				3.1	*ospR*	transcriptional regulator
PA2862			5.3		*lipA*	lactonizing lipase
PA2880			12.2			transmembrane pair domain-containing protein
PA2881			3.2			two-component response regulator
PA2938	4.4	9.5				probable transporter
PA2943	3.6	3.8				phospho-2-dehydro-3-deoxyheptonate aldolase
PA3014				3.5	*faoA*	fatty acid oxidation complex subunit alpha
PA3038	28.1	29.9			*opdQ*	probable porin
PA3064			3.9		*pelA*	biofilm formation protein PelA
PA3233	3.9	6.3				putative signal-transduction protein
PA3234	11.5	25.3			*yjcG*	probable sodium
PA3235	12.5	37.6			*yjcH*	conserved hypothetical protein
PA3271				4.1		two-component sensor
PA3280			4.2		*oprO*	pyrophosphate-specific outer membrane porin OprO

PA3296			6.3		*phoA*	alkaline phosphatase
PA3356	3.3	3.3			*pauA5*	Glutamylpolyamine synthetase
PA3369				5.9		hypothetical protein
PA3370				7.3		hypothetical protein
PA3371				5.0		hypothetical protein
PA3381			3.2			transcriptional regulator
PA3382			7.2		*phnE*	phosphonate transporter PhnE
PA3383			8.0			phosphonate ABC transporter substrate-binding protein
PA3384			4.3		*phnC*	phosphonate ABC transporter ATP-binding protein
PA3389	3.5	12.7				probable ring-cleaving dioxygenase
PA3390	6.6	22.0				putative antibiotic biosynthesis monooxygenase
PA3509			3.5			hydrolase
PA3513			3.4			putative ABC transporter substrate-binding protein
PA3514			5.0			ABC transporter ATP-binding protein
PA3560				4.1	*fruA*	PTS system fructose-specific transporter subunit IIBC
PA3561				4.7	*fruK*	1-phosphofructokinase
PA3562	3.0	4.0			*fruI*	phosphotransferase system transporter enzyme I, FruI
PA3578	3.9	3.0				phenazine biosynthesis PhzC/PhzF protein
PA3690	10.2	14.6				metal-transporting P-type ATPase
PA3766	3.1	2.9				probable aromatic amino acid transporter
PA3779	4.9	10.1				putative periplasmic substrate binding protein
PA3780	3.2	11.6				putative C4-dicarboxylate transporter small subunit
PA3781				5.0		transporter
PA3836			2.9			putative ABC-type transport protein
PA3841	5.2	4.6			*exoS*	exoenzyme S
PA3842	3.8	3.1			*spcS*	specific Pseudomonas chaperone for ExoS, SpcS
PA3901			6.3		*fecA*	Fe(III) dicitrate transporter FecA
PA3970			6.0		*amn*	AMP nucleosidase
PA4022	5.7	4.3			*hdhA*	hydrazone dehydrogenase, HdhA
PA4023	5.0	4.8			*eat*	ethanolamine transporter, Eat
PA4024	3.9	3.3			*eutB*	ethanolamine-ammonia lyase, large subunit, EutB
PA4025			3.4		*eutC*	ethanolamine ammonia-lyase small subunit
PA4054				3.4	*ribB*	3,4-dihydroxy-2-butanone 4-phosphate synthase/GTP cyclohydrolase II-like protein
PA4055	3.1	3.6			*ribC*	riboflavin synthase subunit alpha
PA4073				2.8		aldehyde dehydrogenase
PA4138	3.0	4.0			*tyrS*	tyrosine-tRNA ligase
PA4198	3.9	4.6				acyl-CoA synthetase
PA4199	3.0	3.4				acyl-CoA dehydrogenase
PA4230			3.3		*pchB*	salicylate biosynthesis protein PchB
PA4289				5.3		chromate transporter
PA4290	4.0	29.7				chemotaxis transducer
PA4385				3.5	*groEL*	molecular chaperone GroEL
PA4498	8.5	8.1			*mdpA*	metallo-dipeptidase aeruginosa, MdpA regulated by PsdR
PA4500	4.9	4.5			*dppA3*	probable binding protein component of ABC transporter, regulated by PsdR
PA4501	6.7	6.6			*opdP*	Glycine-glutamate dipeptide porin OpdP
PA4502	5.6	6.1			*dppA4*	probable binding protein component of ABC transporter
PA4503	3.6	3.1			*dppB*	dipeptide ABC transporter permease DppB
PA4504	5.1	4.8			*dppC*	dipeptide ABC transporter permease DppC
PA4505	5.2	4.8			*dppD*	dipeptide ABC transporter ATP-binding protein DppD
PA4506	5.3	4.1			*dppF*	dipeptide ABC transporter ATP-binding protein DppF
PA4542				3.0	*clpB*	chaperone protein ClpB
PA4550	4.0	3.2			*fimU*	type 4 fimbrial biogenesis protein FimU
PA4551	4.4	3.4			*pilV*	type 4 fimbrial biogenesis protein PilV
PA4552			3.4		*pilW*	type 4 fimbrial biogenesis protein PilW
PA4618				4.8		putative xanthine dehydrogenase accessory factor
PA4619				8.3		cytochrome C
PA4620				14.0		putative oxidoreductase
PA4621				22.2		oxidoreductase
PA4625			4.1		*cdrA*	cyclic diguanylate-regulated TPS partner A CdrA
PA4629				4.4		hypothetical protein
PA4635	8.2	7.0			*mgtC*	putative magnesium transporter, MgtC family protein
PA4635a				3.0		hypothetical protein
PA4640				2.9	*mqoB*	malate:quinone oxidoreductase
PA4738				4.7	*yjbJ*	hypothetical protein
PA4739				4.7		transport-associated protein
PA4754				3.1		major facilitator superfamily permease
PA4761				3.2	*dnaK*	molecular chaperone DnaK
PA4821				3.4	*dinF*	transporter
PA4822	11.0	26.1				putative Na+/phosphate symporter
PA4823	21.3	34.4				hypothetical protein

PA4824	47.5	84.1				hypothetical protein
PA4825	28.0	95.3			*mgtA*	Mg(2+) transport ATPase, P-type 2
PA4826	11.9	16.0				hypothetical protein
PA4911			3.1			branched-chain amino acid ABC transporter permease
PA4919				2.9	*pncB1*	nicotinate phosphoribosyltransferase
PA4920				4.4	*nadE*	NAD synthetase
PA4978	3.6	4.3				putative acyl-CoA synthetase
PA4979	3.8	3.3				acyl-CoA dehydrogenase
PA4980	3.4	3.7				enoyl-CoA hydratase
PA4983	4.0	3.3			*dmsR*	probable two-component response regulator
PA4985	13.7	8.7				putative extracellular solute-binding protein
PA4986	3.8	3.5				oxidoreductase
PA5029				11.4	*ynfL*	transcriptional regulator
PA5054				3.4	*hslU*	ATP-dependent protease ATP-binding subunit HslU
PA5081	9.7	4.5				putative hydrolase
PA5082	61.0	19.2				ABC transporter
PA5083	50.9	26.6			*dguB*	Rid2 subfamily protein
PA5084	46.9	22.1				oxidoreductase
PA5153	5.3	3.0				amino acid ABC transporter substrate-binding protein
PA5154	4.4	3.4				ABC transporter permease
PA5167	4.0	15.4			*dctP*	C4-dicarboxylate-binding periplasmic protein DctP
PA5168	3.0	11.1			*dctQ*	tripartite ATP-independent periplasmic transporter DctQ
PA5169				10.7	*dctM*	C4-dicarboxylate transporter
PA5198			3.4		*ldcA*	lysine-specific pyridoxal 5'-phosphate-dependent carboxylase LdcA
PA5302	3.0	12.5			*dadX*	catabolic alanine racemase
PA5303	2.9	9.7				putative endoribonuclease
PA5304	4.1	14.1			*dadA*	D-amino acid dehydrogenase, small subunit
PA5309			3.1		*pauB4*	oxidoreductase
PA5312	4.8	6.6			*pauC*	aldehyde dehydrogenase
PA5313	3.9	5.5			*gabT2*	Transaminase
PA5314	3.6	4.5				putative transcriptional regulator
PA5348			5.4			DNA-binding protein
PA5360				4.1	*phoB*	two-component response regulator PhoB
PA5365				3.5	*phoU*	phosphate uptake regulatory protein PhoU
PA5366				4.1	*pstB*	phosphate ABC transporter ATP-binding protein
PA5367				7.3	*pstA*	phosphate ABC transporter permease
PA5368				6.5	*pstC*	phosphate ABC transporter permease
PA5369	4.5	15.5			*pstS*	phosphate ABC transporter, periplasmic phosphate-binding protein, PstS
PA5381				2.8		hypothetical protein
PA5435				4.7	*oadA*	pyruvate carboxylase subunit B
PA5436				5.4		acetyl-CoA carboxylase subunit A
PA5442			3.5			diguanylate cyclase/phosphodiesterase
PA5445				8.0	*psecoA*	coenzyme A transferase
PA5446				3.2		hypothetical protein
PA5481				10.5		putative lysozyme inhibitor
PA5482				13.1		hypothetical protein
PA5507				14.9		isochorismatase family protein
PA5508				13.5	*pauA7*	glutamine synthetase
PA5522	3.2	3.2			*pauA6*	glutamine synthetase
PA5523	3.2	3.0				probable aminotransferase
PA5530				13.8		C5-dicarboxylate transporter
PA5545	3.8	3.4				putative glutamate/glutamine-binding protein

[a] Differential gene expression of Populations E and D grown with added AHLs, day 160 vs. day 5.
[b] Locus tag and gene name from pseudomonas.com (1).
