# Supplementary material for: Evolution of the Quorum Sensing Regulon in Cooperating Populations of Pseudomonas aeruginosa
Source: mBio. 2022 Feb 22;13(1):e00161-22. doi: 10.1128/mbio.00161-22 (PMC8863103; doi:10.1128/mbio.00161-22)
Supplement: TABLE S4 [file mbio.00161-22-st004.pdf]

Table S4. Strains used in this study

Bacterial strain	Description	Source
P. aeruginosa		
MPAO1	Wild-type *P. aeruginosa*	(2)
D1	Isolated from Population D after 160 passages in CAB	This work
D2	Isolated from Population D after 160 passages in CAB	This work
E1	Isolated from Population E after 160 passages in CAB	This work
E2	Isolated from Population E after 160 passages in CAB	This work
NES24	PqsR (MvfR) S36N variant in MPAO1 background	This work
E. coli		
DH5α	*fhuA2 Δ(argF-lacZ)U169 phoA glnV44 Φ80 Δ(lacZ)M15 gyrA96 recA1 relA1 endA1 thi-1 hsdR17*	Invitrogen
S17-1	*thi pr, hdsR hdsM+ rec,* RP4-2 (Tc::Mu Km::Tn7)	(3)
