# Supplementary material for: Evolution of the Quorum Sensing Regulon in Cooperating Populations of Pseudomonas aeruginosa
Source: mBio. 2022 Feb 22;13(1):e00161-22. doi: 10.1128/mbio.00161-22 (PMC8863103; doi:10.1128/mbio.00161-22)
Supplement: TABLE S5 [file mbio.00161-22-st005.pdf]

Table S5. Plasmids and primers used in this study

Plasmids	Relevant characteristics	Source
pMAL-t-*aiiA*	Maltose binding protein-AiiA fusion vector, Ap^{R}	(4)
pPROBE-GT	Source for multiple cloning site (mcs) *gfp* fusion, Gm^{R}	(5)
pBBR1MCS-5	Broad-host range plasmid, Gm^{R}	(6)
pBBR-*gfp*	Promoterless *gfp* transcriptional reporter; pBBR1MCS with mcs, *gfp* fusion from pPROBE-GT, Gm^{R}	This work
pP$_{pqsA}$-*gfp*	pBBR-*gfp* with -429 to 0 of the *pqsA* promoter, Gm^{R}	This work
pEXG2	Allelic exchange vector with pBR origin, *sacB*, Gm^{R}	(7)
pNS12	pEXG2 containing sequences to create the PqsR S36N variant allelic exchange vector, Gm^{R}	This work

Primer	DNA sequence (5' to 3')	Description
psdR_F	GGGTTCGGGTAGTTCATC	*psdR* SNP detection
psdR_R	GTTTGCCTGACAGGATGG	*psdR* SNP detection
mcsGFPpBBR_F	ATCGGTGCGGGCCTCTTCGCTATTACGCCAGCAAGCT TGCATGCCTGCAGGTC	pBBR-gfp construction
GFP_pBBR_R	CTAAAGGGAACAAAAGCTGGGTACCCTATTTGTATAGT TCATCCATGCCATGTGTAATCC	pBBR-gfp construction
vec_pBBRGFP_F	ATGGCATGGATGAACTATACAAATAGGGTACCCAGCTT TTGTTCCCTTTAGTGAG	pBBR-gfp construction
vec_pBBRGFP_R	ATCCTCTAGAGTCGACCTGCAGGCATGCAAGCTTGCT GGCGTAATAGCGAAGAGG	pBBR-gfp construction
PpqsA_pBBR_F	GGGCCTCTTCGCTATTACGCCAGCAAGATGCCGTCGC CCCCTTGGAG	pP$_{pqsA}$-*gfp* construction
PpqsA_pBBR_R	ATCCTCTAGAGTCGACCTGCAGGCATGCAAGCATGAC AGAACGTTCCCTCTTCAGC	pP$_{pqsA}$-*gfp* construction
vec_pBBRpqsA_F	TATCGCTGAAGAGGGAACGTTCTGTCATGCTTGCATGC CTGCAGGTCGAC	pP$_{pqsA}$-*gfp* construction
vec_pBBRpqsA_R	TGGGCTCCAAGGGGGGCGACGGCATCTTGCTGGCGTA ATAGCGAAGAGG	pP$_{pqsA}$-*gfp* construction
pqsR_SNP1_F	ATTGCAACTGGTCTATTTTCCTCTTATGCTGGTTGCCG AAACGGGCCATC	pNS12 construction
pqsR_SNP2_R	TGCTGACCGCCGAGtTGACCGCGGTGTG	pNS12 construction
pqsR_SNP3_F	TCGCACACCGCGGTCAaCTCGGCGGTCAGCAACCTG	pNS12 construction
pqsR_SNP4_R	TCCTTTTATGATTTTCTATCAAACAATTCCATCCCGAGT CGATTCTCACCACCCACGGCCA	pNS12 construction
pEXG2-pqsRS36N_F	GCCGTGGGTGGTGAGAATCGACTCGGGATGGAATTGT TTGATAGAAAATCATAAAGGATTTG	pNS12 construction
pEXG2-pqsRS36N_R	TCGCTGGAGATGGCCCGTTTCGGCAACCAGCATAAGA GGAAAATAGACCAG	pNS12 construction

Ap^{R}, resistant to ampicillin; Gm^{R}, resistant to gentamicin

References

1. Winsor GL, Griffiths EJ, Lo R, Dhillon BK, Shay JA, Brinkman FS. 2016. Enhanced annotations and features for comparing thousands of *Pseudomonas* genomes in the Pseudomonas genome database. Nucleic Acids Res 44:D646-53.
2. Jacobs MA, Alwood A, Thaipisuttikul I, Spencer D, Haugen E, Ernst S, Will O, Kaul R, Raymond C, Levy R, Chun-Rong L, Guenthner D, Bovee D, Olson MV, Manoil C. 2003. Comprehensive transposon mutant library of *Pseudomonas aeruginosa*. Proc Natl Acad Sci U S A 100:14339-44.
3. Simon R, Priefer U, Puhler A. 1983. A broad host range mobilization system for in vivo genetic engineering: transposon mutagenesis in gram negative bacteria. Bio/Technology 1:784-791.
4. Thomas PW, Fast W. 2011. Heterologous overexpression, purification, and in vitro characterization of AHL lactonases. Methods Mol Biol 692:275-90.
5. Miller WG, Leveau JH, Lindow SE. 2000. Improved *gfp* and *inaZ* broad-host-range promoter-probe vectors. Mol Plant Microbe Interact 13:1243-50.
6. Kovach ME, Elzer PH, Hill DS, Robertson GT, Farris MA, Roop RM, 2nd, Peterson KM. 1995. Four new derivatives of the broad-host-range cloning vector pBBR1MCS, carrying different antibiotic-resistance cassettes. Gene 166:175-6.
7. Rietsch A, Vallet-Gely I, Dove SL, Mekalanos JJ. 2005. ExsE, a secreted regulator of type III secretion genes in *Pseudomonas aeruginosa*. Proc Natl Acad Sci U S A 102:8006-11.
